# The invasive cell coat at the microsporidian *Trachipleistophora hominis*–host cell interface contains secreted hexokinases

**DOI:** 10.1002/mbo3.696

**Published:** 2018-07-27

**Authors:** Sophie Ferguson, John Lucocq

**Affiliations:** ^1^ Structural Cell Biology Group School of Medicine University of St Andrews St Andrews UK

**Keywords:** cell coat, hexokinase, immuno‐EM, microsporidia

## Abstract

Microsporidia are obligate intracellular parasites causing significant disease in humans and economically important animals. In parallel to their extreme genetic reduction, Microsporidia have evolved novel mechanisms for exploiting host metabolism. A number of microsporidians confer secretion of otherwise cytosolic proteins by coding for signal peptides that direct entry into the endoplasmic reticulum. The human pathogen *Trachipleistophora hominis* encodes for four hexokinases, three of which have signal peptides at the N‐terminus. Here, we localized hexokinase 2 and hexokinase 3 through developmental stages of *T. hominis* using light and electron microscopy. Both proteins were concentrated in an extracellular coat previously termed the plaque matrix (PQM). The PQM (containing hexokinases) was morphologically dynamic, infiltrating the host cytoplasm predominantly during replicative stages. Throughout development the PQM interacted closely with endoplasmic reticulum that was demonstrated to be active in membrane protein biosynthesis and export. The impact of hexokinase on the host metabolism was probed using the fluorescent analog of glucose, 2‐NBDG, which displayed spatially restricted increases in signal intensity at the parasite/vacuole surface, coincident with hexokinase/PQM distribution. Gross metabolic aberrations, measured using metabolic profiling with the Seahorse XF Analyzer, were not detectable in mixed stage cocultures. Overall, these results highlight a role for the extended cell coat of *T. hominis* in host–parasite interactions, within which secreted hexokinases may work as part of a metabolic machine to increase glycolytic capacity or ATP generation close to the parasite surface.

## 1 INTRODUCTION

Microsporidia are a diverse group of obligate intracellular eukaryotic parasites that comprise over one thousand named species infecting a wide range of eukaryotic cells. These organisms produce significant disease in economically important animals, and can cause life‐threatening infections in immunosuppressed or immunodeficient humans (Bromenshenk et al., 2010; Didier & Weiss, 2011; Stentiford et al., 2016). Clinical studies have shown that the prevalence of microsporidia infections in immunocompetent individuals can be substantial (Anane & Attouchi, 2010; Sak, Kváč, Kučerová, Květoňová, & Saková, 2011).

Formerly classified as early offshoots in eukaryotic evolution (Hirt et al., 1999), microsporidians are now considered to be related to fungi (Karpov et al., 2014; Lee et al., 2008, 2010). Importantly, they display extreme degrees of genetic reduction resulting in incomplete metabolism that is particularly evident in ATP‐generating pathways (Wiredu Boakye et al., 2017). Accordingly, microsporidians display a variable reduction in glycolytic pathways which is combined with significant loss of mitochondrial functions including oxidative phosphorylation. Indeed a striking result of reductive evolution is the relict mitochondrial organelle, the mitosome, which has but a single known residual function—iron sulfur cluster assembly (Freibert et al., 2017; Goldberg et al., 2008).

In parallel to the evolutionary loss of metabolic pathways, microsporidians are hypothesized to have evolved diverse and novel strategies for exploiting host cell metabolism. Mechanisms already described include the expression of multiple nucleotide transporter proteins (NTTs) for ATP uptake at the parasite plasma membrane (Heinz et al., 2014) and direct binding of host mitochondria to the parasite vacuole combined with clustering of mitochondrial outer membrane channel for ATP (voltage‐dependent anion complex; VDAC) at the vacuole membrane (Hacker, Howell, Bhella, & Lucocq, 2014).

Another emerging strategy for exploiting the host appears to be secretion of factors into the host cell cytoplasm (Campbell, Williams, & Yousuf, 2013) with a substantial fraction of exported parasite proteins coding for signal peptides that can direct them into the endoplasmic reticulum for export from the cell (Cuomo et al., 2012; Heinz et al., 2012). Therefore, the secretomes of a number of parasites are now of intense interest, and one comprehensive analysis of Microsporidia‐derived proteins in *Caenorhabditis elegans* revealed 82 parasite‐derived proteins at the host–parasite interface, including two that entered the host cell nucleus (Reinke, Balla, Bennett, & Troemel, 2017). Several signal peptide‐containing microsporidian proteins also contain leucine‐rich repeats known to act as pathogenicity factors in fungi (Butler et al., 2009; Campbell et al., 2013), while a number of versions of the normally cytosolic glycolytic enzyme hexokinase (HK) have been shown to contain signal peptides (Cuomo et al., 2012; Heinz et al., 2012). In five microsporidian species, these HKs containing signal peptides have been shown to successfully enter and navigate a yeast secretory pathway (Cuomo et al., 2012), with a total 11 sequenced genomes showing signal peptide motifs in HK sequences. The *C. elegans* study also detected a secreted HK in the cytoplasm, while the *Antonospora locustae* isoform has been localized to the nucleus (Reinke et al., 2017; Senderskiy, Timofeev, Seliverstova, Pavlova, & Dolgikh, 2014). However, the precise high‐resolution localization and function of the HKs in each of these settings remain unclear.

HKs catalyze phosphorylation of glucose to glucose‐6‐phosphate, therefore secreted HKs could have the potential to drive glycolysis in the host for metabolic advantage. For example, one possibility is the manipulation of metabolism, pushing cells toward a cancer‐like phenotype, the Warburg effect—an aerobic hyper‐glycolytic, apoptosis‐resistant, and anabolic phenotype of cancer. This metabolic state results not only in production of ATP but also in the supply of carbon metabolites for increased biomass, as well as apoptosis avoidance by HK‐VDAC binding, all of which could favor parasite growth (Hsu & Sabatini, 2008; Pastorino & Hoek, 2008).

Here we tested the hypothesis that secreted microsporidian hexokinases work at the host–parasite interface to manipulate glucose usage and/or delivery of energy metabolites. We localized two HKs with genes coding for signal peptides from the microsporidian *T. hominis* using immunofluorescence and electron microscopy and found them concentrated in a cell coat, previously designated as the Plaque Matrix (PQM; Weidner, Canning, & Hollister, 1997). The PQM appears as an amorphous electron dense structure lying at the interface between the parasite or parasite vacuole and host cell cytoplasm and is similar to structures described in several other microsporidians (Desjardins et al., 2015; Fries et al., 1999; Karthikeyan & Sudhakaran, 2016; Vávra & Becnel, 2007; Vávra, Horák, Modrý, Lukeš, & Koudela, 2006). In *T. hominis* we found the PQM (Weidner et al., 1997) becomes infiltrative during rapidly growing vegetative stages before forming part of the sporophorous vacuole structure and interacts extensively with the host cell endoplasmic reticulum throughout the parasite life cycle. Additionally, the PQM was associated with enrichment of the glucose analog 2‐NBDG close to the parasite/vacuole surface. Thus, our results identify a HK‐rich extended cell coat of *T. hominis* with a putative function in manipulating host cell glucose metabolism and/or energy substrate delivery.

## 2 MATERIALS AND METHODS

### 2.1 Cell culture

Rabbit kidney cells (RK13; obtained from the Embley group, University of Newcastle) infected with *T. hominis* were maintained in normal growth medium (MEM GlutaMAX (Gibco, Thermo Scientific, MA, USA) supplemented with 10% (v/v) FCS, 100 U/ml penicillin/streptomycin, 100 μg/ml kanamycin, 1 μg/ml fungizone) at 35°C, 5% CO_2_/95% air.

### 2.2 Light microscopy

Light microscopy techniques were performed at room temperature unless otherwise specified. Cells were grown in 6 well plates on 22 × 22 mm coverslips until 70–90% confluent and fixed in either 4% paraformaldehyde (PFA) in phosphate‐buffered saline (PBS) at room temperature (HK3) or methanol acetone (1:1 v/v) at ‐20°C (HK2). After fixation, coverslips were washed (PBS; 3 × 5 min) and aldehyde fixed cells were permeabilized using 0.1% triton x‐100 (5 min). The coverslips were incubated on fetal calf serum (FCS; 5%) followed by the primary antibody, (HK2 (1:200) and HK3 (1:500) antiserum diluted in 5% FCS; raised against custom peptides, 21^st^ Century Biochemicals) for 1 hr. After washes in PBS (3 × 5 min), coverslips were incubated with fluorescent secondary antibody (1:750; goat α‐rabbit; Thermo Scientific, MA, USA) in the dark for 1 hr. Cell nuclei were counterstained with DAPI (500 ng/ml; 10 min), and following washes with PBS (3 × 5 min) and ddH_2_O (2 × 1 min), coverslips were mounted on glass slides with ProLong Gold antifade reagent (Thermo Scientific, MA, USA).

For structural studies, Giemsa stain (improved R66 solution; VWR Chemicals) was prepared as a 5% v/v solution in pH 7.2 buffer and passed through a 0.22 μm Millipore filter to clear debris. Cells were grown on 22 × 22 mm coverslips and fixed in 4% PFA in PBS (30 min). They were permeabilized using 0.2% v/v Triton x‐100 (5 min) and incubated with stain for 45 min before rinsing twice in ddH_2_O, air drying and mounting in ProLong Gold antifade reagent (Thermo Scientific, MA, USA).

### 2.3 Immuno‐EM

RK13 cells infected with *T. hominis* were grown in 75 cm^2^ flasks and fixed at room temperature in 10 ml 0.5% glutaraldehyde in 0.2 M PIPES buffer (pH 7.4). Cells were scraped into 1 ml fixative and centrifuged to form a pellet. The fixative was removed and the pellet washed with PBS before overnight 4°C incubation in 2.1 M sucrose. The sample was mounted on a specimen holder and plunge‐frozen in liquid nitrogen. 70 nm sections were cut at −100°C using a Leica EM FC7 cryo‐ultra‐microtome and retrieved using 1:1 (v/v) 2% methyl cellulose: 2.1 M sucrose pick‐up solution, before mounting on copper EM grids coated with carbon/pioloform films.

Grids, with sections attached, were stored in dried‐down pick‐up solution, which was removed by floating grids on ice cold ddH_2_O (3 × 5 min). Subsequent labeling steps were then performed at room temperature and comprised the following: droplets of PBS (1 min), 0.5% fish skin gelatin (FSG; 10 min), primary antibodies diluted in 0.5% FSG (HK2 = 1:50, HK3 = 1:100; 30 min), washes in PBS (3 × 5 min), and incubation on protein A gold (1:50 in FSG; 20 min; BBI). After washes in PBS (6 × 5 min), and then ddH_2_O (10 × 1 min), contrasting was performed using uranyl acetate (3%)/methylcellulose (2%) (1:9 v/v) and 0.2 mm gauge tungsten wire loops, as described previously (Griffiths et al., 1984). Sections were imaged in a JEOL 1200EX transmission electron microscope at 80 keV, equipped with a Gatan Orius 2k × 2k CCD digital camera. Images were captured using Gatan Digital Micrograph software.

### 2.4 Quantification of gold labeling

Scanning band analysis (Watt, Kular, Fleming, Downes, & Lucocq, 2002; Lucocq et al., 2004) was performed at the microscope at 5,000× magnification. A band was created on the live camera display and comprised a defined guard area for organelle identification, an upper acceptance line and a lower forbidden line. A single point was defined in the middle of the band to trace out a line probe and count membrane intercepts during scanning. As the sample was translocated, gold particles were selected according to the forbidden line counting rule, assigned to cellular compartments and intercepts with organelle membranes counted. For quantification of label over PQM, images were taken of all parasites/parasite groups at 1,500–3,000× magnification and gold counted live at the microscope, ignoring profiles repeated in subsequent sections. For host nuclei, images were captured systematic uniform random (Lucocq, 2012) at ×5,000 magnification and imported into ImageJ (NIH, USA). A randomly placed square lattice stereology grid (Mironov, 2017) was applied for estimation of area by point counting and gold density calculated.

### 2.5 Epoxy resin embedding

Cells were grown in a 75 cm^2^ flask and fixed with 0.5% glutaraldehyde/0.2 M PIPES (pH 7.4), scraped, and centrifuged to form a pellet. The fixative was removed, and cell pellets were postfixed in 1% osmium tetroxide containing 1.5% potassium ferrocyanide in 0.1 M sodium cacodylate buffer, pH 7.4. Pellets were then washed in 0.1 M sodium cacodylate pH 7.4 (×3) and dehydrated in graded ethanols (30–95%, 10 min each), followed by 100% ethanol (2 × 20 min), propylene oxide (2 x 5 min) and embedding in Durcupan resin (Sigma Aldrich, MO, USA). Sections, 50–80 nm thick, were then cut on a diamond knife and mounted on 200 hexagonal mesh or 2 mm slot grids (serial sections), coated with pioloform and stained with Reynolds lead citrate.

### 2.6 Glucose‐6‐phosphatase cytochemistry

All reactions were performed at room temperature. Cells, grown to 70–90% confluency in a 10 cm^2^ dish, were fixed in 0.5% glutaraldehyde/0.2 M PIPES (pH 7.4) buffer for 30 min and washed in 0.2 M PIPES buffer (pH 7.4; 3 × 5 min) before permeabilization in 0.2 M PIPES (pH 7.4)/10% v/v DMSO for 3 days at 4°C. A reaction mixture was formed by adding 12% w/v lead nitrate dropwise into a glass vial of 80 mM Tris maleate (pH 6.5) containing 10% v/v DMSO (Tris‐DMSO) with stirring, to achieve a final concentration of 0.096% lead nitrate. After addition of each drop, the solution was allowed to clear. The fixed cell monolayer was washed in Tris‐DMSO, then incubated with gentle agitation for 2 hr in reaction mixture containing 19 mg/ml glucose‐6‐phophate. Cells were then washed twice in Tris‐DMSO, scraped into 1 ml of sodium cacodylate buffer and centrifuged at 16,100*g* for 5 min in a plastic microfuge tube to form a pellet. The supernatant was removed, and a 1% BSA solution in 0.2 M PIPES used to resuspend the pellet. An equal volume of 1% glutaraldehyde in 0.2 M PIPES was added, and the resulting suspension centrifuged for 20 min at 16,100*g*. The pellet was stored under fixative for epoxy resin embedding (see above).

### 2.7 Expression of GFP‐ts045‐VSVG

*Trachipleistophora hominis* infected RK13 cells were transfected with the construct (GFP‐ts045‐VSVG pCDNA3.1) using Lipofectamine 2000 reagent (Thermo Scientific, MA, USA). For each well of a six‐well dish, 2.5 μg DNA was mixed with 6 μl Lipofectamine 2000 diluted in Opti‐MEM Media (Thermo Scientific, MA, USA), incubating at room temperature for 5 min, and applied to cells before incubation at 35°C, 5% CO_2_/95% air overnight. Next day, cultures were moved to 40°C for 4 hr to accumulate GFP‐ts045‐VSVG in the ER and then moved to 32°C for 0, 30, and 75 min to allow protein refolding and export into the secretory pathway. Coverslips were fixed (4% PFA) and counterstained using immunofluorescence of the Golgi marker, GLG1 (1:200; Abcam, Cambridge, UK; as described above for HK2 and HK3).

### 2.8 Seahorse XFp analyzer glycolytic stress assays

The instrument, all consumables and analysis programs were obtained from Seahorse Bioscience (Agilent), MA, USA. Cells were seeded into eight‐well Seahorse XFp assay plates at a density of 70%–90% confluence in normal growth media (see Cell Culture). These were left overnight (35°C, 5% CO_2_/95% air) prior to assay. Next day, media was exchanged for glycolysis stress test media (DMEM, 2 mM glutamine) and placed at 37°C 100% air for 1 hr before conducting assays in the Seahorse XFp Analyzer. The Glycolysis stress test involved sequential injections of glucose (10 mM final concentration), oligomycin (1 μM), and 2‐deoxyglucose (50 mM). Postassay, cells were lysed in radioimmunoprecipitation lysis buffer (RIPA; 1 × Roche protease inhibitors) and protein assays (BCA) conducted to normalize results. Data were analyzed using Seahorse Report Generator.

### 2.9 2‐NBDG and calcein AM assay

ThRK13 cells were seeded onto 35 mm fluorodishes (World Precision Instruments, FL, USA) in normal growth media (see Cell Culture). Next day, cells were incubated for 2.5 hr (35°C, 5% CO_2_) with Opti‐MEM/200 μM 2‐NBDG in DMSO/100 μM Hoescht 33342 (Thermo Scientific, MA, USA). Cells were treated with Opti‐MEM/Calcein AM 2.5/100 μM Hoescht 33342 and incubated for 1 hr (35°C, 5% CO_2_). Cells were then washed and media replaced with Ham's F12 media (Gibco, Thermo Scientific, MA, USA). Dishes were sealed and cells imaged using the Nikon N‐SIM Microscope system mounted with a 37°C stage, fitted with an iXon + 897 EMCCD camera (Andor Technology, Ulster, UK).

Images were assessed quantitatively using Image J. Gray level values were plotted along 3 μm long linear intercepts that were drawn radially in a direction from the center of each cell profile. As the lines crossed the cell periphery orthogonally they were positions so that 1.5 μm of the line was on the inside of the cell and 1.5 μm was the outside. For each cell profile, five intercepts were measured at angles selected systematic random but only recorded when the cell periphery was adjacent to host cytoplasm and not when other parasites were present. Each local gray value was expressed as a fraction of the density range for that intercept ranging from 0 to 1 (mean density value, Figure 11).

## 3 RESULTS

### 3.1 Hexokinases of *T. hominis* are evolutionarily conserved, metabolically active and encode signal peptides

We analyzed the protein sequences of *T. hominis* HKs, identified as already described (Wiredu Boakye et al., 2017). In this paper, we identify four: HK1 (THOM_0443), HK2 (THOM_0898), HK3 (THOM_0937), and HK4 (THOM_1847‐8), of which three (HK1, 2, and 3) have N‐terminal signal peptides identified using software SignalP and PrediSi (Hiller, Grote, Scheer, Münch, & Jahn, 2004; Petersen, Brunak, von Heijne, & Nielsen, 2011). Two of these genes, HK2 and HK3, were investigated further. Of these, HK2 appears to have a misannotated start codon, identified by trimming 20 residues to the next methionine that lies next to a strong signal peptide sequence.

Comparison to solved structures of *Saccharomyces cerevisiae* HK PII (HXK2) (Kuser, Krauchenco, Antunes, & Polikarpov, 2000) revealed conservation of high proportions of functional residues, involved in catalysis, substrate binding, and turnover, as well as enzyme flexibility. As previously reported (Wiredu Boakye et al., 2017), functional residues in the *S. cerevisiae* HXK1 (conserved in HXK2 also) active site such as Asp^211^ (catalytic base), Thr^234^ and Ser ^419^ (ATP binding), Glu^269^ and Glu^302^ (glucose binding) are all highly conserved in the *T. hominis* isoforms HK2 and 3. In addition, we found a high degree of conservation of residues involved in enzyme flexibility (glycines) and of charge in the putative hydrophobic channel (see Table S1). These data strongly indicate the conservation of enzymatic function across divergent organisms.

### 3.2 Hexokinases 2 and 3 localize to juxta‐parasite region of the host cytoplasm

**Figure 1:**
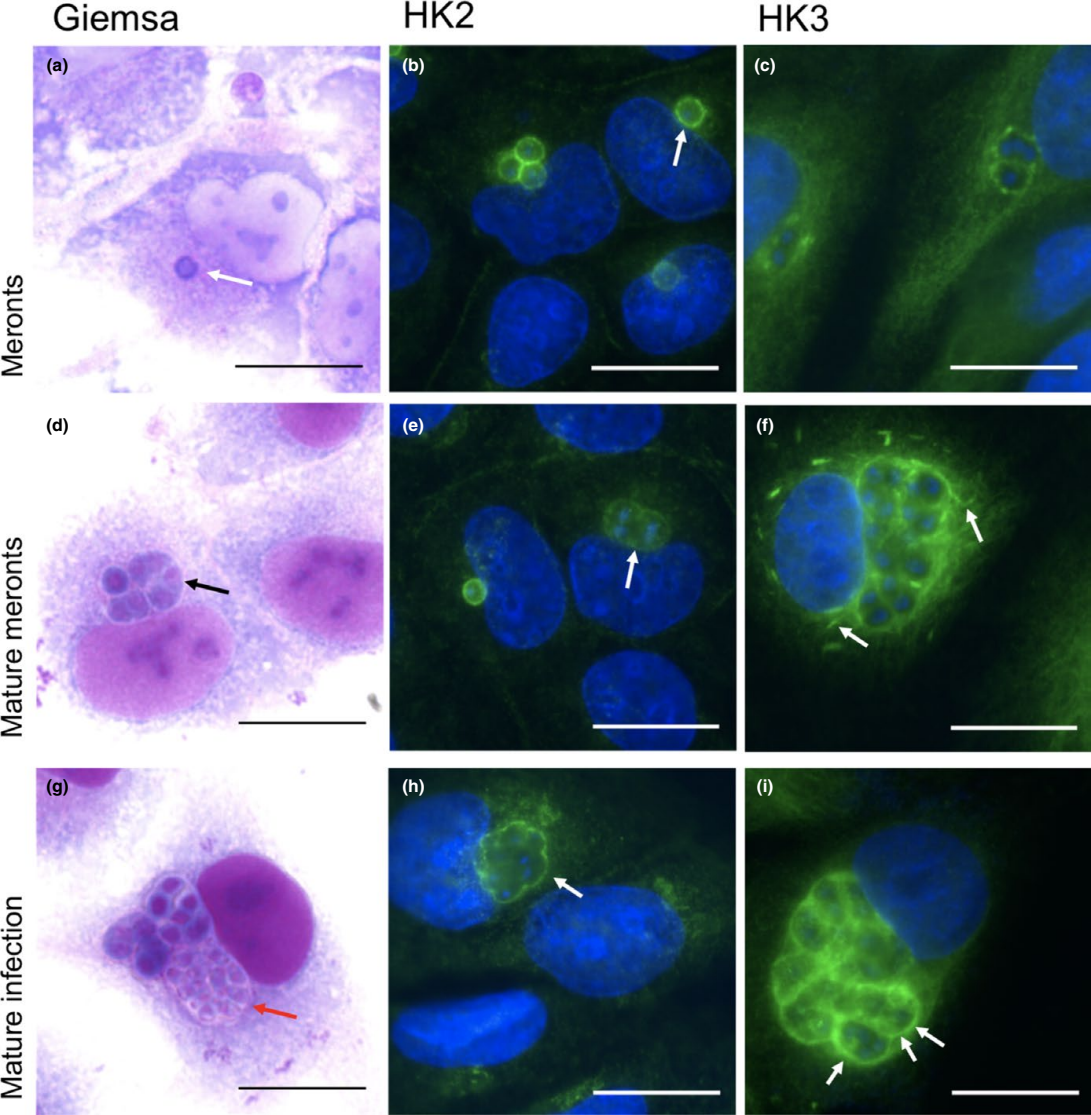
Immunofluorescence localization of HK2 and HK3 in *Trachipleistophora hominis*‐infected monolayers of RK13 cells. Leishman Giemsa staining (a, d and g; PFA fixation) provides an overview of parasite ontogeny showing a single early meront (a; white arrow), a mature meront (d; coenocytium black arrow), and mature infection with cellularized sporonts and spores (g; red arrow; stain is largely excluded from later parasite stages). Staining with HK2 antiserum (b, e, and h; methanol acetone fixation) shows weak staining over the cytoplasm of meronts, but strong linear staining pattern restricted to the periphery of parasites and parasite groups (arrows). The strongest peripheral staining is associated with early meronts (b), decreasing in intensity through multinucleate and spore forming stages. Immunofluorescence staining with the HK3 antiserum (c, f, and i; PFA fixation) displays a similar pattern to HK2. Weakest peripheral staining was seen in single early meronts (c) with stronger staining seen through multinucleate stages and after cellularization (f and i (arrows)). HK3 staining often extended into the cytoplasm around the parasite periphery (arrows in f). Bars = 15 μm

**Figure 2:**
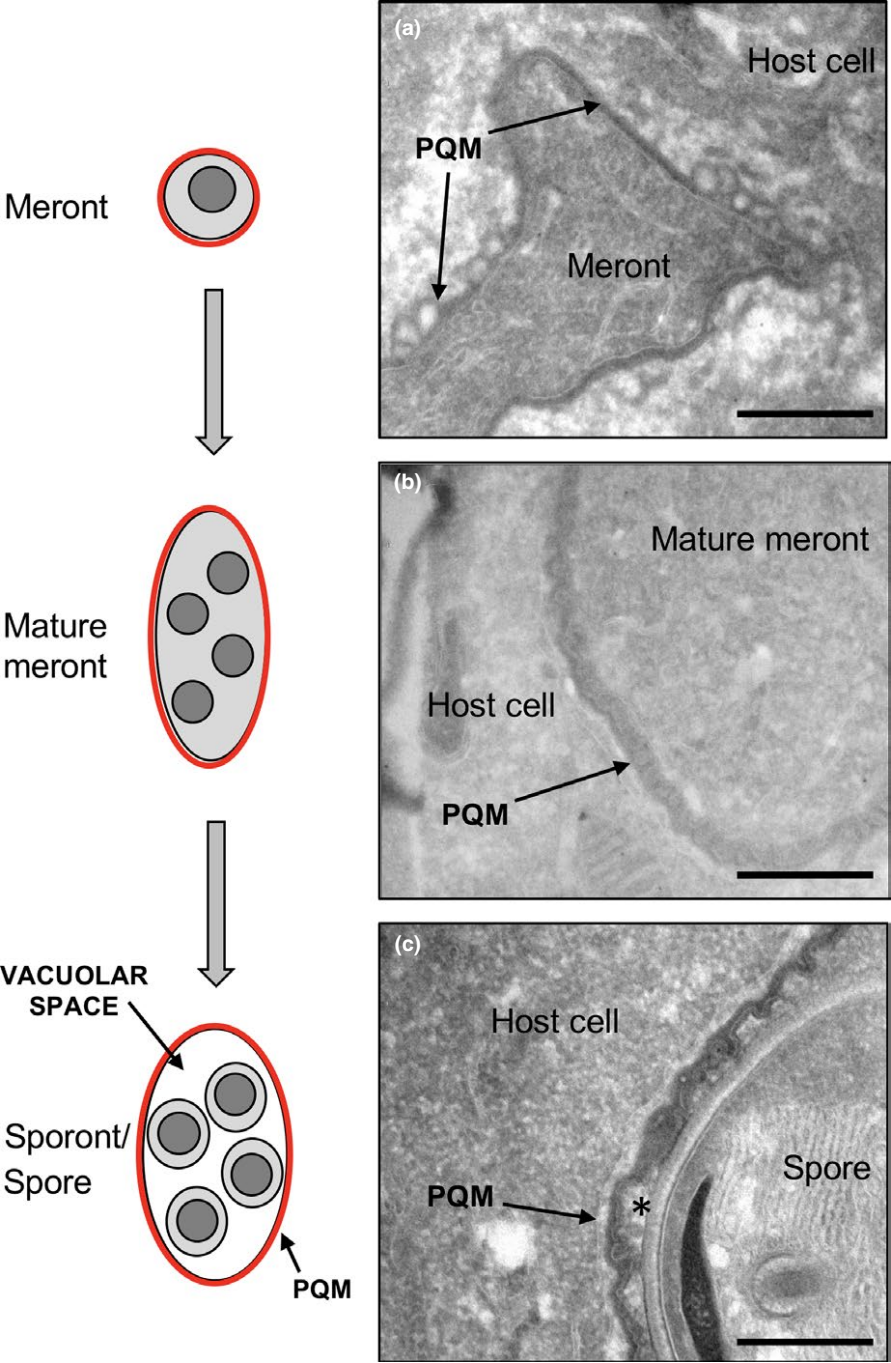
Ultrastructure changes in the parasite periphery and plaque matrix (PQM) through maturation. Replication and development of *T. hominis* begins with a single meront in direct contact with the host cytoplasm (a). Single meronts undergo nuclear division without cytokinesis, resulting in a coenocytic stage, termed here the mature meront (b). Parasites then undergo cellularization, forming sporonts situated inside a sporophorous vacuole. The spore wall and infection apparatus develop, and parasites reach full maturation as the infectious stage (c). During the meront stages (a and b), the PQM coats the surface of the parasite, with infiltration of the host cell cytoplasm. The PQM of mature meronts encloses no discernible vacuolar space, but becomes more complex in structure, with a membrane‐like internal structure appearing ruffled in cross‐section (b and c; see Figure 3). After cellularization and the development of the cell wall, a vacuolar space (asterisk in c) appears between sporonts/spores and the PQM forms the wall of the sporophorous vacuole (SV). Additionally, the membrane‐like structure of the PQM (now constituting the sporophorous vacuole) appears to become partially deconvoluted. Bars = 500 nm

Immediately post infection in the RK13 cell culture system, *T. hominisy* appear as single nucleated meronts located in the peripheral host cytoplasm (Figures 1a and 2a). Single meronts appear to undergo full mitosis which is followed by multiple nuclear divisions to form a plasmodial coenocytium. These then cellularize prior to maturation into infective spores (Figure 1a, d and g) (Hollister et al., 1996). Cellularization is accompanied by formation of a sporophorous vacuole (SV), which surrounds groups of developing spores (see Figures 1 and 2).

Peptide antibodies raised against HKs were verified against bacterially expressed protein (Western blotting; Figure S1) and used for localization studies (HK1 was not investigated further because antibodies raised against this ortholog did not provide consistent signals in immunofluorescence after aldehyde fixation).

Immunofluorescence staining of HK2 revealed striking peripheral staining at the interface between the parasite and the host cytoplasm throughout most developmental stages. The strongest staining occurred around single parasites at early postinfective vegetative stages (meronts; Figure 1b). Through further development, the staining was restricted to the periphery of groups of proliferating nuclei (Figure 1e), and also groups of mature spores (Figure 1h)—consistent with localization to the SV or its immediate vicinity. Throughout development staining for HK2 also occasionally extended further into the host cell cytoplasm in a dendritic, finger‐like fashion (not shown). HK3 was also found in the host cell cytoplasm but displayed some key differences. In early stages (single and double cells), the staining was discontinuous and extended further into the host cell cytoplasm than HK2 (Figure 1c). In later development (coenocytic forms (mature meronts)), the signal became more linear with some short segments of staining apparently disconnected from the main body of HK3 staining around the parasites (Figure 1f). In late maturation, HK3 staining appeared more restricted to the periphery of parasite groups (Figure 1i). These results prompted us to undertake ultrastructural investigations of the host–parasite interface.

### 3.3 Electron microscopy reveals the complexity of the cell coat (PQM) at the host cell interface

**Figure 3:**
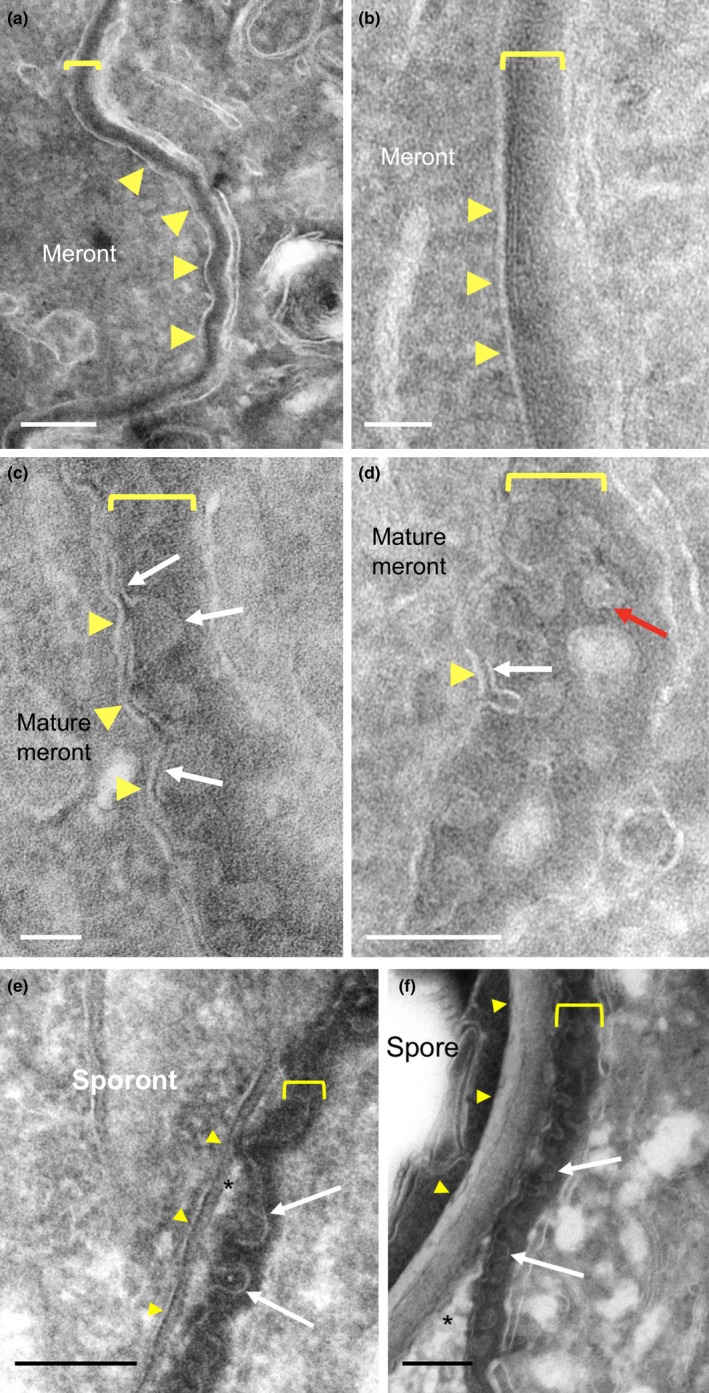
Interfacial structures between *T. hominis* parasites and host RK13 cells visualized in Tokuyasu thawed frozen sections. In early meronts (a and b), plasma membranes of parasites (arrowheads) are coated with an amorphous electron dense coat (bracketed). In orthogonal sections, the coat displays linear features (b) but no consistent internal membrane‐like structures. At multinucleate (mature meront) stages (c and d), membrane‐like structures appear within the coat (arrows) and display outpocketings that with a branched “bunches of grapes” pattern (red arrow in d). The PQM coat is intimately and consistently associated with cisternal elements of host cell membranes (see text and Figure 9). After cellularization (sporonts and spores; e and f respectively), the plasma membrane of the parasite (arrowheads) has become separated from the coat by the cell wall and surrounding vacuolar space (asterisk). The coat (brackets) with internal membrane‐like structure now surrounds the group of cellularized parasites (sporonts) forming the sporophorous vacuole. Host cell membranes remain closely associated with the coat (f). Bars = (a, e, f) 200 nm, (b–d) 50 nm

**Figure 4:**
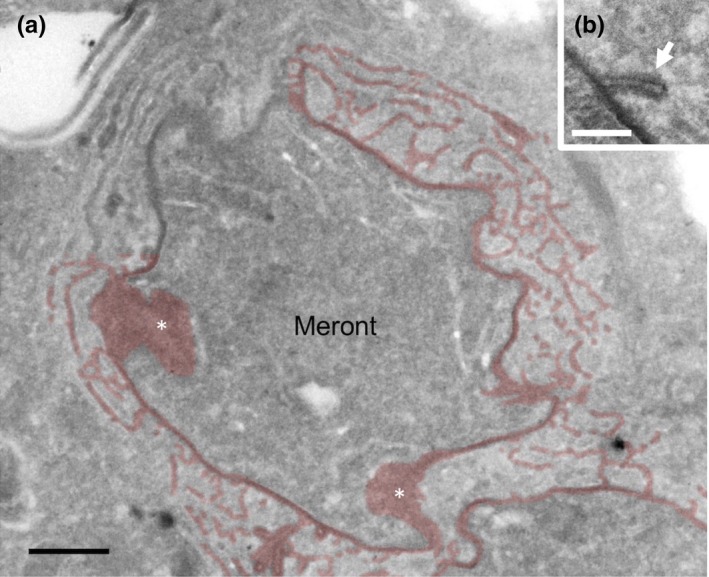
Extensions of the cell coat (PQM) into the host cell cytoplasm. (a) The cell coat of meronts displays protrusions of similar thickness and density to the cell coat (PQM) immediately surrounding the parasites. The parasite coat and extensions are pseudo‐colored to highlight the extent of host cytoplasm infiltration at low magnification, with the region top‐left uncolored for comparison. White asterisks indicate parasite surface (PQM structures) tangentially sectioned. (b) Rod‐like structures with electron lucent interior extend from Athe parasite coat. Image is from a Tokuyasu thawed frozen section. Bars = (a) 500 nm, (b) 200 nm

The detailed structure of the local parasite environment was investigated using thawed frozen sections (Tokuyasu technique) and conventional epoxy resin sections in transmission electron microscopy. In *T. hominis* infections, the cell boundary is not defined by a simple plasma membrane structure. Throughout parasite development, we identified an amorphous electron dense layer which was uniformly present (Figures 2, 3 and 4). This has been described previously as the Plaque Matrix (PQM; (Weidner et al., 1997)) and throughout this report we adopt this terminology.

The development of the parasites, described above, is summarized (Figure 2, left panel) and compared to the ultrastructure of the PQM (Figure 2a–c). This method provided superior display of the amorphous PQM and allowed measurement orthogonal to the parasite membrane (42.2 ± 3.9 nm, *N* = 3, from 58, 18, and 34 micrographs, respectively). Morphologically, in early meronts, the PQM appeared as a smooth and uniform coat over parasite plasma membrane with extensions of the structure into the host cell cytoplasm (Figure 4; see below). Additionally, when investigated at higher magnification, linear features running parallel to the underlying plasma membrane were visible in orthogonal sections (Figure 3b).

With further parasite maturation the PQM appeared to increase in thickness with development of an internal membrane‐like structure that was uniformly crenated in profile (Figures 3c‐f), likely reflecting knob‐like structures rather than ridges in 3D. Postcellularization, the PQM (with its internal membrane‐like structure) became separated from the parasite cell surface, forming a vacuolar space (Figures 2c and 3e and f). Throughout development host cell membranes were closely associated with the outer aspect of the PQM (see below).

### 3.4 Plaque matrix (PQM) infiltrates the host cell cytoplasm during parasite replication

Infiltration of PQM into the host cell cytoplasm was a marked feature of early meronts (Figure 4) with extensions connecting over the entire meront surface (Figure 4a). Some of the infiltrative elements contained characteristic unbranched rod‐like structures, some of which were found to connect to the surface PQM (Figure 4b). These occurred singly or in groups (see below) and displayed a tubular morphology. The amorphous components of extended PQM were analyzed further using serial sections, revealing extensively interconnected loops, fingers, or plate‐like morphologies joining to the surface PQM (Figure S2a–f) and occasionally seen to connect between discrete meronts (Figure S2d–f and g). Like the PQM at the parasite surface, the extended PQM was also coated with host cell membrane compartment(s) (see below).

### 3.5 HK2 and HK3 are concentrated in the PQM

**Figure 5:**
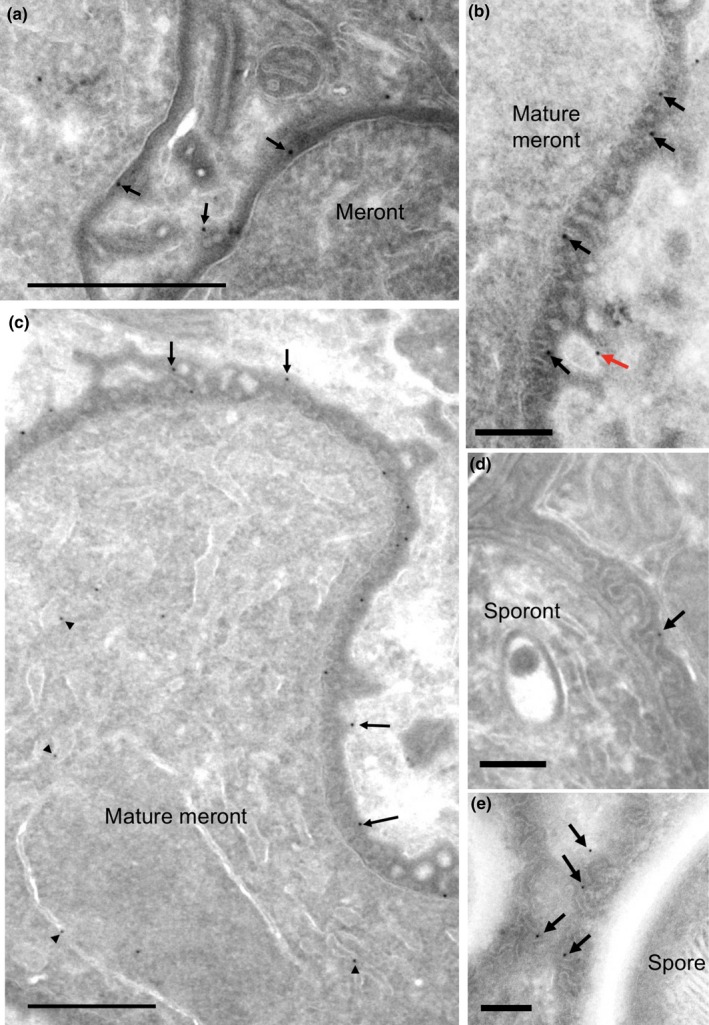
Immunoelectron microscopy of HK2. HK2 antiserum was applied to Tokuyasu cryosections of *T. hominis* infected RK13 cells and localized using protein A gold (10 nm) as described in the Materials and Methods. In early meronts (a), mature meronts (b and c), sporonts (d), and spores (e) labeling of parasites was largely located over cell coat (PQM; arrows) and also over parasite intracellular membranes and nuclear envelope (arrowheads in (c)). Some protrusions of the cell coat (PQM) into the host cell cytoplasm were also labeled (b; red arrow). Bars = (a, c) 500 nm, (b, d, e) 200 nm

**Figure 6:**
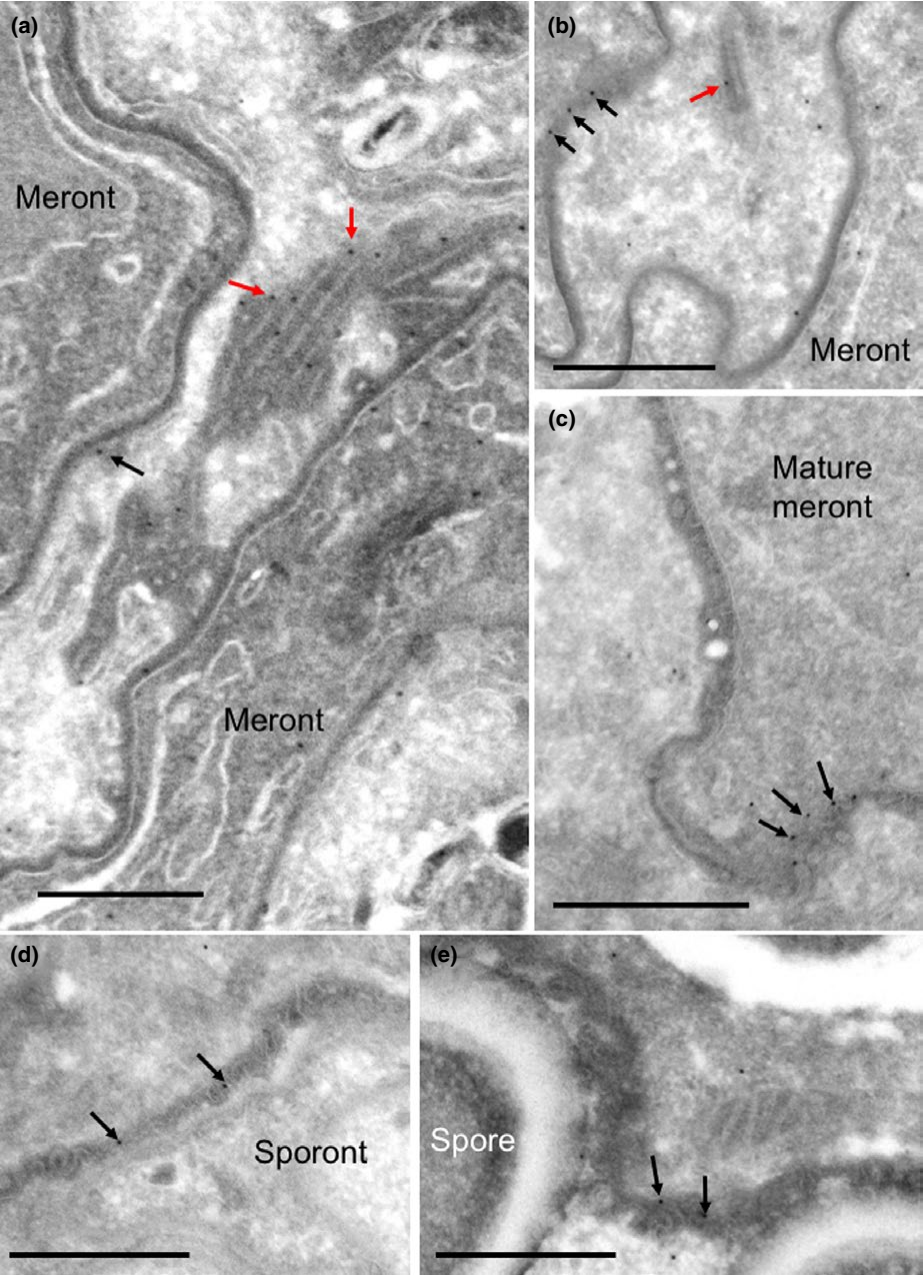
Immunoelectron microscopy of HK3. Tokuyasu cryosections of *T. hominis* infected RK13 cells were prepared as described in the Materials and Methods and incubated with rabbit antiserum raised against HK3 followed by 10 nm protein A gold. Labeling of the cell surface PQM (black arrows) was present in early meronts (a and b), mature meronts (c), sporonts (d), and spores (e). Labeling over cell coat extensions into the host cytoplasm was present at meront stages and was also associated with rod‐like structures (a and b; red arrows). Bars = 500 nm

To correlate the immunolocalization of secreted HKs with high‐resolution structure, the Tokuyasu thawed frozen section method was combined with immunogold labeling (Figures 5 and 6). Inside early and mature meronts, HK2 labeling was observed over internal parasite membranes morphologically identifiable as ER (decorated with ribosomes), nuclear envelope and putative Golgi tubules (Figure 5a and c; (Beznoussenko et al., 2007)). Outside the meront, gold labeling was observed over the surface PQM and its extensions into the host cell cytoplasm (Figure 5a and b). Labeling was also observed in the same distribution after cellularization and SV formation (Figure 5d and e). The distribution of labeling for HK3 was similar to that for HK2, although the labeling over the cytoplasmic extensions of the PQM appeared more marked. Labeling for HK3 was also found over regions of the PQM interspersed between the rod‐like tubular components (Figure 6a and b). As with HK2, labeling for HK3 was present over the PQM consistently throughout parasite maturation (Figure 6). Qualitatively, there was no concentration of labeling over the host compartments for either isoform.

### 3.6 Quantification of immunogold labeling for HK2 and HK3

**Figure 7:**
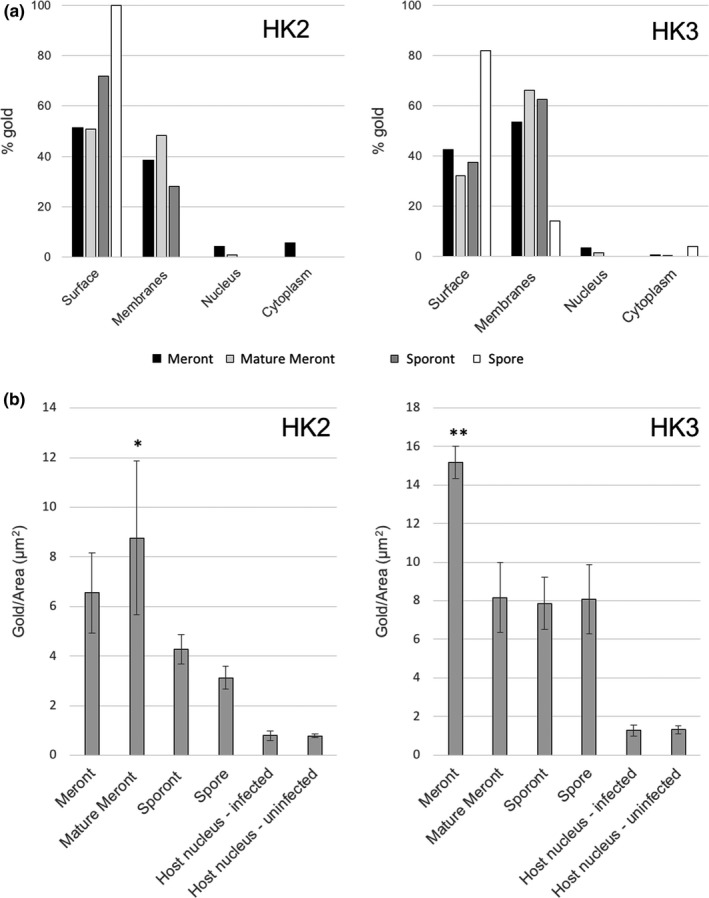
Quantification of immunogold labeling for HK2 and HK3s in parasites. (a) Distribution of gold labeling. Labeled thawed cryosections were imaged “live” using a digital camera and gold particles counted in a systematic array of scans that was randomly positioned (scanning band analysis; see Materials and Methods). “Surface” labeling includes that associated with the cell membrane coat and the extensions of the coat into the host cell cytoplasm. The majority of labeling was associated with the coat and the parasite intracellular membranes (“Membranes”), most of which display tubulovesicular or cisternal profiles, and likely correspond to ER and Golgi. (b) Labeling intensity. For parasite compartments, images of whole parasite profiles were captured and areas estimated using point counting. For host nuclei, sets of systematic micrographs were taken over parasite profiles and areas (estimated using point counting) related to gold counts in the same micrographs. The labeling over PQM was most concentrated in the meront phases of development and was consistently more intense than over the host cell nucleus (both infected and uninfected). Statistical analysis consisted of Kruskal–Wallis test with Dunn's multiple comparison test. *p < 0.05 versus host nucleus (infected). *^*^ *p* < 0.01 versus host nucleus (infected). *N* = 3. Error bars = ±*SD*

Initially, scanning band analysis (detailed in Materials and Methods, (Watt, Kular, Fleming, Downes, & Lucocq, 2002; Lucocq et al., 2004)) was performed to investigate the distribution of gold particles (summarized in Figure 7a). Labeling for both isoforms demonstrated enrichment over the surface (constituting the PQM and underlying plasma membrane) and concentration of label over internal membrane structures of *T. hominis* (more marked for HK3 than HK2). The concentration over these membranes, some of which can be morphologically identified as rough ER, is consistent with trafficking through the secretory pathway.

To quantify the intensity of labeling, we analyzed whole parasite profiles and host nuclei in micrographs sampled systematic uniform random (SUR) using stereological principles (see Materials and Methods; Figure 7b). The concentration of gold over PQM at all stages of development was consistently higher than the control compartments from nucleated cell profiles of either infected or uninfected cells. Using the nucleus of infected cells as a reference compartment, there was a significant concentration of gold label for HK2 over the mature meront PQM (*p* < 0.05, *N* = 3; 11‐fold more concentrated), and for HK3 over the meront PQM (*p* < 0.01, *N* = 3; 12‐fold more concentrated).

We next analyzed immunogold labeling for evidence that secreted HK was localized to other host cell compartments other than the PQM (Figure S3). There was no apparent concentration of HK labeling over the cytosolic aspect of membrane‐bound compartments/organelles of the host cell (Figure S3). In particular, we did not find evidence for an increase in labeling over the mitochondrial outer membrane. This argues against binding of HK to the outer mitochondrial membrane porin, VDAC and a role for HKs in conferring apoptosis resistance or aerobic glycolysis at that site.

**Figure 8:**
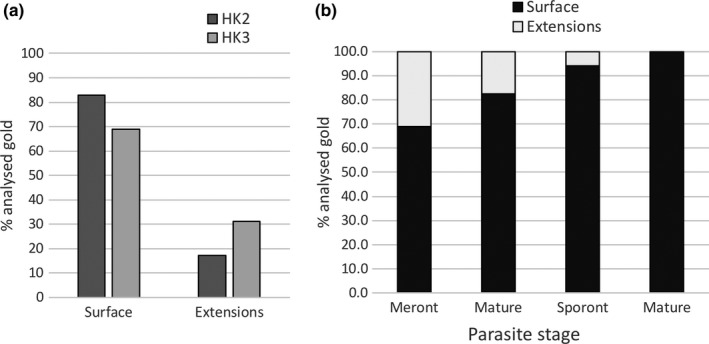
Evaluation of HK2 and HK3 labeling over parasite surface PQM and cytoplasmic extensions. (a) Gold was categorized as surface (within a single particle width of the surface‐associated PQM) or extensions (labeling PQM extending into the host cytoplasm). Gold particles associated with tangentially sectioned PQM without an orthogonal underlying membrane, were excluded due to ambiguity. Compared to HK2, HK3 showed a greater proportion of label over PQM extensions. (b) Further analysis of HK3 labeling revealed the proportion of label over PQM extensions diminished with parasite maturation, most likely due to the retraction or involution of infiltrative PQM elements

Further analysis of HK2 and HK3 localization over PQM elements revealed subtle differences. Specifically, a larger proportion of HK3 labeling was associated with infiltrative PQM compared to HK2 (31.1% vs. 17.1%; Figure 8a). This is consistent with the more filamentous nature of HK3 immunofluorescence at the interface with the host cell cytoplasm (Figure 1f). Additionally, when examining *T. hominis* ontogeny, the proportion of labeling over PQM extensions diminished with maturation of the parasite, correlating with the lessening infiltration by PQM in later stages (Figure 8b).

### 3.7 PQM‐associated host cell membranes are biosynthetically active ER

**Figure 9:**
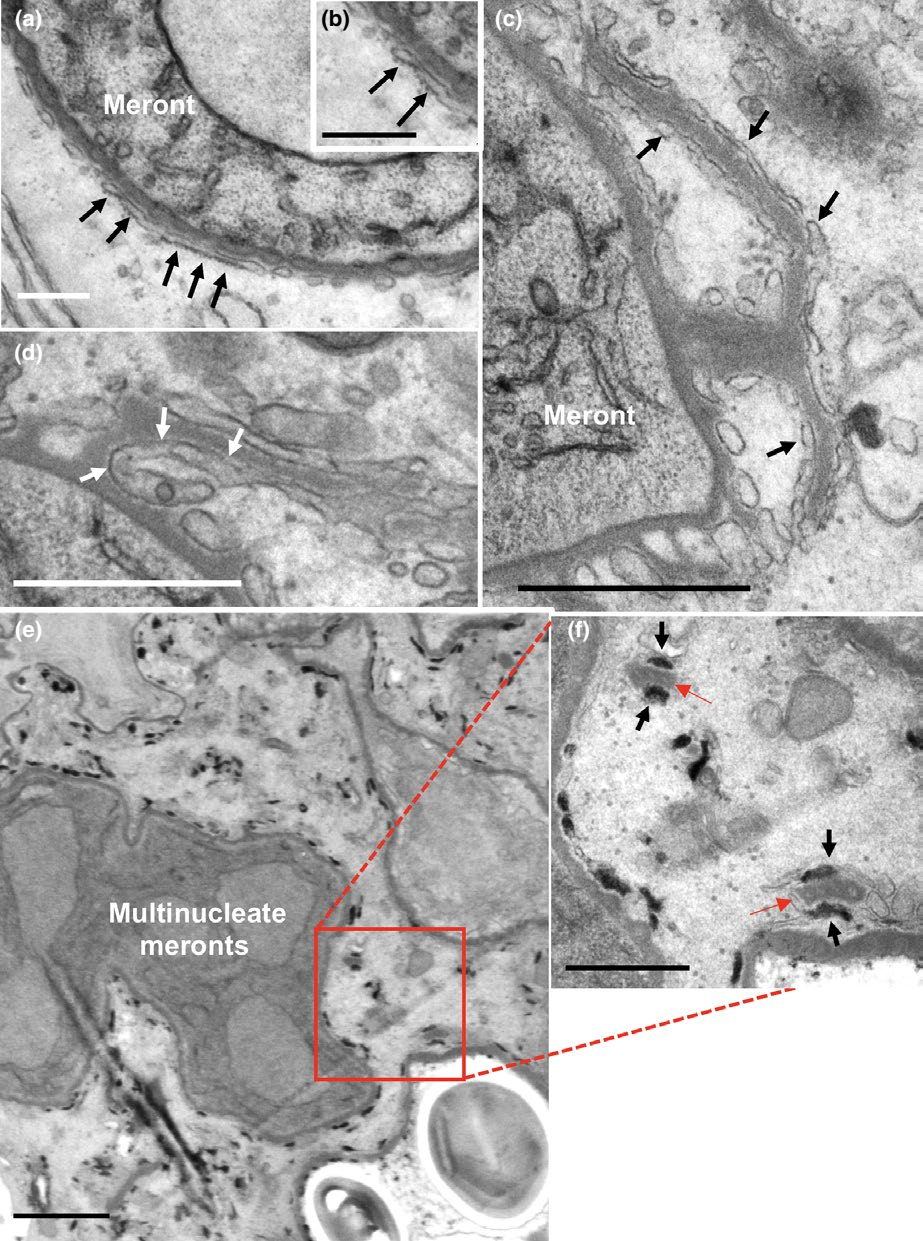
Host endoplasmic reticulum is extensively associated with *T. hominis* PQM at the parasite surface and over cytoplasmic extensions. In epoxy embedded samples (for improved membrane contrast compared to cryosections), cisternal/tubular profiles consistently decorate the PQM. Cisternae situated over the parasite surface coat are decorated with ribosomes (arrows in a and inset b). The association of cisternal/tubular membranes with the coat is extensive, and also involves extensions of the PQM from the cell surface components (c (arrows) and d). Arrows in d indicate the continuity between membranes covering the surface coat and those covering the coat extensions. The cytochemical reaction for G6P‐ase activity, a luminal ER marker, showed clear electron dense staining in the lumen of membrane compartments, associated with both surface and extended PQM (e overview and f high magnification; G6Pase reaction product—black arrows; PQM—red arrows). Bars = (a, c, d, f) 500 nm, (b) 200 nm, (e) 1 μm

Membranes associated with PQM were analyzed morphologically using convention epoxy resin embedded material and transmission electron microscopy. These membranes were associated with both surface and extended PQM, with surface PQM membranes displaying predominantly cisternal‐like morphology, and those associated with extended PQM being more tubulovesicular in appearance (Figure 9a–d). A substantial proportion of membranes displayed bound ribosomes, especially the cisternal‐like elements covering PQM at the parasite surface (Figure 9a and b). Both membrane components appeared extensively interconnected at all stages of parasite development (Figure 9c and d).

To further investigate the identity of PQM‐associated membranes, the cytochemical reaction for the luminal ER marker glucose‐6‐phosphatase was used (Figure 9e and f). Reaction product was observed in the lumen of cisternal and tubulovesicular membrane‐bound structures, associated with both surface PQM and its extensions. The smooth membranes of extended PQM can therefore be classified as smooth ER.

**Figure 10:**
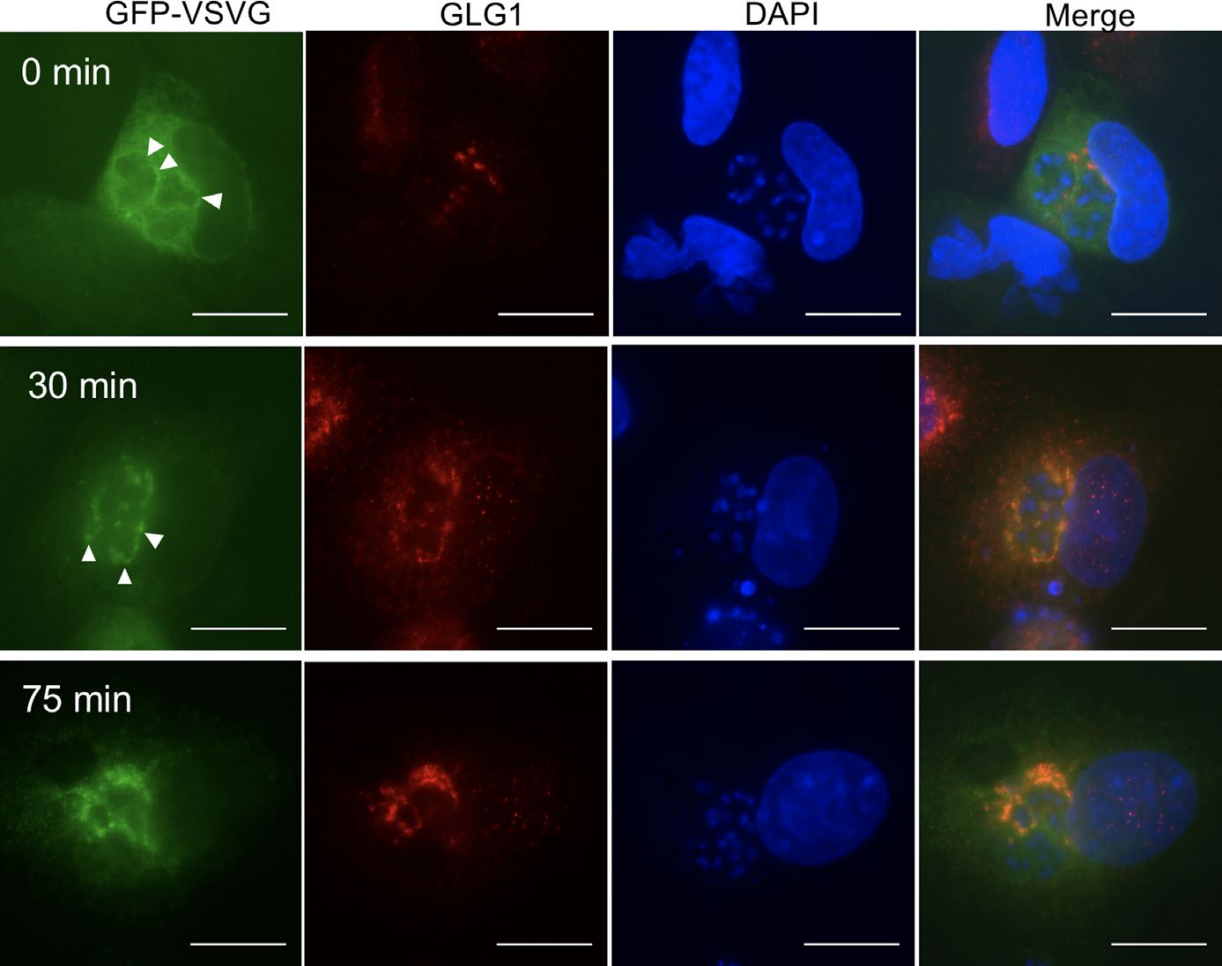
PQM‐associated host ER is biosynthetically active. Expression of the GFP conjugated viral glycoprotein, ts045‐VSVG, provides a tool for monitoring the activity in the secretory pathway. Cells were transfected, then incubated at 40°C for 4 hr to accumulate misfolded GFP‐tsO45‐VSVG in the ER lumen (0 min), before shifting to 32°C for the given time periods to monitor traffic (30 and 75 min). Cells were then fixed and stained for the Golgi marker, GLG1 (red). Immediately after accumulation (0 min), GFP‐tsO45‐VSVG fluorescence can be seen throughout the cytoplasmic ER and nuclear envelope of host cells and is concentrated at the periphery of parasite groups (arrowheads). After 30 min at 32°C, the ER signal has decreased and the staining co‐localizes with GLG1. At 75 min, the colocalization with GLG1 is maintained, but additional punctate signals are scattered in the cytoplasm, consistent with staining of vesicles destined for the plasma membrane. Bars = 15 μm

To test whether PQM‐associated ER membranes retained biosynthetic capability, we expressed a GFP‐tagged temperature‐sensitive mutant of vesicular stomatitis virus G protein (GFP‐tsO45‐VSVG; Figure 10; (Scales, Pepperkok, & Kreis, 1997; Presley et al., 1997)). At the restrictive temperature (40°C), the protein misfolds and accumulates in the ER lumen, whereas shifting the cells to the permissive temperature (32°C) allows correct folding and export from the ER. At 40°C (4 hr), fluorescence was concentrated in the nuclear envelope of host cells and in the region immediately surrounding groups of parasite nuclei. After 30 min at 32°C, fluorescence colocalized with the Golgi marker (Golgi glycoprotein 1, GLG1; (Kawano et al., 2002)), while after further incubation at 32°C (75 min) GFP fluorescence was more widely dissemination in the cell with the appearance of punctate vesicle‐like structures. Comparison of infected with uninfected cells demonstrated comparable GFP‐VSVG cellular distributions, suggesting that infection does not affect the ER export rate (Figure S4).

### 3.8 Functional assessment of metabolism in *T. hominis* infected cells

Our localization studies indicated a role for HKs in host cell metabolism, such as upregulation of glucose turnover and/or exploitation of pathways for energy production. Initial studies used Seahorse XFp technology to investigate global metabolism in *T. hominis* infected cultures. This system measures real‐time flux of glycolysis and oxidative phosphorylation under different metabolic stresses, assessing the impact of *T. hominis* on these metabolic pathways. Specifically, the technology detects production of lactate as measure of glycolysis, although it cannot assess glucose uptake (Teslaa & Teitell, 2014). We could not detect consistent changes in glycolysis (Figure S5a) or oxidative phosphorylation (Figure S5b) using this method in *T. hominis* infected cultures. One reason for is that the proportion of cells infected was 7.64% (±2.90; *N* = 4). We tried to increase the proportion of cells by mixing concentrated spore preparations with already infected RK13 cells without success.

**Figure 11:**
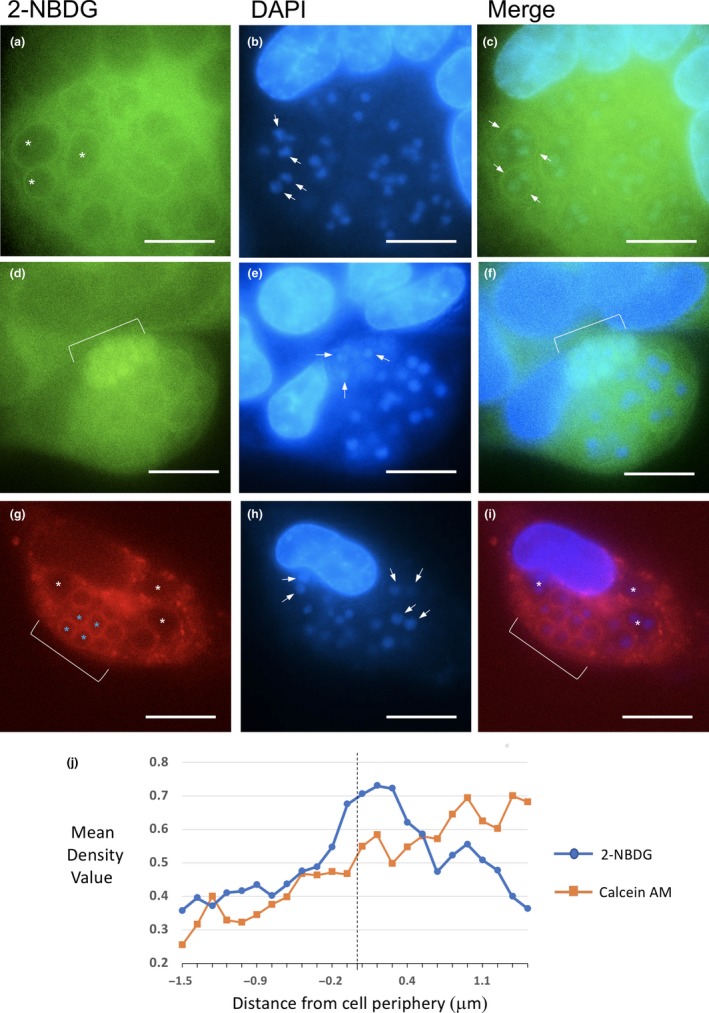
The glucose analog 2‐NBDG concentrates around multinucleate parasites. T. hominis‐infected RK13 cells were incubated for 2.5 hr with 200 μM 2‐NBDG, and imaged live to investigate the distribution of intracellular glucose/G6P. Multinucleate meronts (a–c) were identified by groups of nuclei (arrows in b) and larger surrounding cytoplasm (asterisks in a and arrows for nuclei in b) and show a linear accumulation of 2‐NBDG (arrows in c). Intense accumulation of signal was also seen inside the cytoplasm of cellularized sporonts/spores, identified by their smaller cytoplasm and smaller nuclei (brackets and arrows in d–f). The cytosolic tracking dye Calcein AM was employed to control for any possible local increase in representation of (organelle poor) cytosolic compartments in this region, leading to a rise increase in local signal. Meront groups (g–i; white asterisks and arrows) appeared negative for linear accumulation around vacuoles. However, Calcein AM did have a stronger signal around later‐stage, cellularized parasites (g–i; brackets and blue asterisks), suggesting interactions with the nascent cell wall. (j) Signals for 2‐NBDG or Calcein AM were quantified along radial linear intercepts straddling the cell periphery as described in the Materials and Methods. Signals for 2‐NBDG show a peak of intensity which extends from the cell periphery (dotted line) into the host cell cytoplasm. N = 5 with mean coefficients of error for all values 8.6% (2‐NBDG) and 7.8% (Calcein AM). Bars (a–i), 10 μm

Another method for assessing glycolysis is the use of glucose analogs such as 2‐[N‐(7‐Nitrobenz‐2‐oxa‐1,3‐diaxol‐4‐yl)amino]‐2deoxy glucose (2‐NBDG; (Teslaa & Teitell, 2014)). This reagent monitors uptake into cells via glucose transporters, is then phosphorylated by HK and subsequently retained within the cell. Importantly, the signal can be visualized by live fluorescence microscopy with the potential to identify local accumulations within the cytosol or intracellular organelles. RK13 cells infected with *T. hominis* were incubated with 200 μM of 2‐NBDG in Opti‐MEM media for 2.5 hr and imaged using wide‐field fluorescence microscopy (Figure 11). We then compared the level of fluorescence between infected and uninfected cells, and carefully assessed the spatial distribution of the signal in the region of the parasite and SV. Overall, there did not appear to be a detectable difference in the uptake of 2‐NBDG between infected and uninfected cells. However, there appeared to be two pools of more intense signal in infected cells. First, there was accumulation of linear signals around groups of replicating meronts (Figure 11a–c and j; white asterisks and arrows) which on quantification appeared to extend into the surrounding cytoplasm (J). Second there was an enrichment within the cytoplasm of spores (Figure 11d–f; arrows).

To control for differences in soluble cytosolic components, the cytosolic tracer dye Calcein AM was employed. This dye has been used to establish cell volume, due to its retention in live cells and homogenous distribution in cytoplasm (Johnson, 1998). It also appears to remain inert, without impacting on cell fitness (Braut‐Boucher et al., 1995). The general pattern of fluorescence of Calcein AM in and around meronts was homogeneous and in marked contrast to the 2‐NBDG signal pattern, displayed no linear pattern (Figure 11g–i; white asterisk and arrows) or comparable peak of intensity (J). An apparent enrichment in the periphery of spores, was consistent with cell wall staining (Figure 11g–j; blue asterisk.

## 4 DISCUSSION

In this report, we document the structure, composition, and function of an amorphous cell coat (previously given the term Plaque Matrix or PQM), that encloses the intracellular forms of the microsporidian *T. hominis*. The PQM is visible at the earliest times after infection and appears to be dynamic, dendritic in form, and infiltrates the host cell cytoplasm during the early stages of parasite development. As cellularization proceeds, the PQM distribution becomes more restricted and it forms part of a specialized vacuole wall structure (the SV) that becomes separated from the parasite plasma membrane. Within the SV‐associated PQM, a second membrane‐like structure develops and encloses the vacuolar space that contains the differentiating spores. Thus, this cell coat is continually present at the host cell interface and is well placed for interaction with the host and exploitation of host cell metabolism.

The PQM is not limited to *T. hominis* and similar structures have been described in a number of microsporidian species (Desjardins et al., 2015; Fries et al., 1999; Karthikeyan & Sudhakaran, 2016; Vávra & Becnel, 2007; Vávra et al., 2006; Weidner et al., 1997). In some reports, infiltration into the host cell cytoplasm has also been described. These observations suggest that a range of microsporidians use similar mechanisms at the host cell interface. Similar coat structures have also been described in a diverse array of other intracellular parasites, including Apicomplexans (De Souza, 1995; Dogga, Bartošová‐Sojková, Lukeš, & Soldati‐Favre, 2015). These cell coats, however, are largely associated with surface adhesion and invasion of the host cell, rather than infiltration of the host cytoplasm. Our localization of two HKs to the cell coat of *T. hominis* represents a first step in assigning a metabolic function to the PQM.

Both HKs contain predicted signal peptides and two strands of evidence suggest they direct entry into the parasite secretory pathway. First, both HKs were localized to the rough ER and vesiculotubular structures within the parasites, indicating synthesis and targeting to the ER are followed by export into the Golgi. Secondly, when HKs were expressed transiently in RK13 host cells (HK3; unpublished), we observed a strong nuclear envelope signal and ER staining pattern, indicating entry into the secretory pathway of these mammalian cells. Thus, while a number of other glycolytic enzymes are known to be secreted by parasitic organisms independent of appropriate signal peptides (Karkowska‐Kuleta & Kozik, 2014), this does not appear to be the case for the two HKs studied here. Using the Microsporidial genome repository, MicrosporidiaDB (Aurrecoechea et al., 2010), the intersection of “hexokinase” and predicted signal peptides (SignalP) shows the presence of corresponding genes in 11 species—*Edhazardia aedis*,* Encephalitozoon cuniculi*,* Encephalitozoon hellem*,* Encephalitozoon intenstinalis*,* Encephalitozoon romaleae*,* Nematocida ausubeli*,* Nematocida parisii*,* Nosema bombycis*,* Nosema ceranae*,* T. hominis*, and *Vavraia culicis*. Furthermore, a secretory trap expression system in *S. cerevisiae*, showed HK signal peptides in *N. parisii, E. cuniculi, Antonospora locustae*, and *N. ceranae* can direct cargo protein into the ER and successfully navigate the yeast secretory pathway (Cuomo et al., 2012). The widespread expression of secreted HKs in the face of genome compaction suggests an essential role. So far, two studies have localized signal peptide‐containing microsporidian HKs to host cell compartments ((Senderskiy et al., 2014), *A. locustae*, nucleus; (Reinke et al., 2017) *N. parisii,* cytoplasm) although they did not establish functions for HKs at these sites.

We show the PQM of *T. hominis* interacts extensively with the ER, decorating both cell‐bound and infiltrative portions of PQM during all parasite stages we examined. We confirmed the identity of these ER elements using cytochemical demonstration of G6P‐ase at the EM level. The consistent presence of the ER over the PQM surface suggests tight binding, since there did not appear to be any substantial areas of the coat that were bare of ER. Interestingly, the parasite surface‐associated PQM‐ER was more often coated by ribosomes while the infiltrating PQM‐associated ER was mostly smooth in appearance. In experiments using the GFP‐ts045‐VSVG, we also confirmed that the PQM‐associated ER functions in biosynthesis of membrane proteins and it is also competent to export this cargo into the nearby Golgi stacks; explaining the occurrence of ER exit sites (EM) and COPII (immunofluorescence of sec13; not shown). Therefore, a substantial portion of the membrane biosynthetic capacity of the host resides in the PQM‐ER complex. It will be of great interest to study proteins and functions involved in PQM‐ER interaction (see below) and determine whether the ER has a positive or negative function in parasite host interactions. The binding of host ER is a documented feature of intracellular parasites such as Legionella or *Toxoplasma gondii*. In Legionella (Robinson & Roy, 2006), the surrounding ER membranes undergo binding and fusion which is dependent on COPII proteins. In Toxoplasmosis, the binding of ER membranes (Sinai, Webster, & Joiner, 1997) and fusion with the parasite vacuole (Melo & de Souza, 1997) relies on the SNARE protein sec22, a process that enables cross‐presentation of parasite located proteins (Goldszmid et al., 2009). In our study, we could not find evidence for mixing of ER proteins with the vacuole contents, either by fluorescence (GFP‐ts045‐VSVG) or by glucose‐6 phosphate EM cytochemistry, but further investigation of the mechanisms of ER association and the possible role of SNAREs in this process would be of interest.

So, what might be the metabolic impact of secreted hexokinases? The simplest scenario is that upregulation of glycolytic pathways in the infected cell produces ATP for import via documented ATP transporters present in the parasite plasma membrane throughout development (Heinz et al., 2014). We tested this possibility, using Seahorse metabolic profiling but could not find evidence for global upregulation of glycolysis (Figure [Link], [Link]). However, these results should be interpreted in the light of low levels of *T. hominis* infection in the RK13 monolayers, and restricted distribution of HK to the PQM. Interestingly we also could not detect an effect on global metabolism in *E. cuniculi* where there was a much higher infection rate. In this organism, the single hexokinase is predicted to navigate the secretory pathway and preliminary analysis in immunofluorescence and immuno‐EM using antibodies raised against this protein (not shown) revealed staining of the cytoplasm and internal membranes and cell periphery without extension into the host cell cytoplasm. As this organism makes no detectable extended coat structure, our interpretation is that any metabolic effect of this hexokinase will be tightly focused at the cell surface of the parasite and would have a minor impact on host cell metabolism.

In contrast, we could detect accumulation of the glucose analog 2‐NBDG (or its phosphorylated form) in a location that coincided with the PQM structure—suggesting glucose processing/metabolism is concentrated at parasite periphery. Thus, HK activity could be driving glycolysis and generating ATP close to the parasite/vacuole, with potential to feed carbon skeletons into macromolecules such as amino acids for the growing parasite (*T. hominis* gene sequences predict amino acid transporters; (Heinz et al., 2012)). If this is true, *T. hominis* would have evolved a mechanism for stealing ATP that is distinct from the clustering of host cell mitochondria documented to occur in other microsporidians (e.g., *E. cuniculi*; (Hacker et al., 2014)).

**Figure 12:**
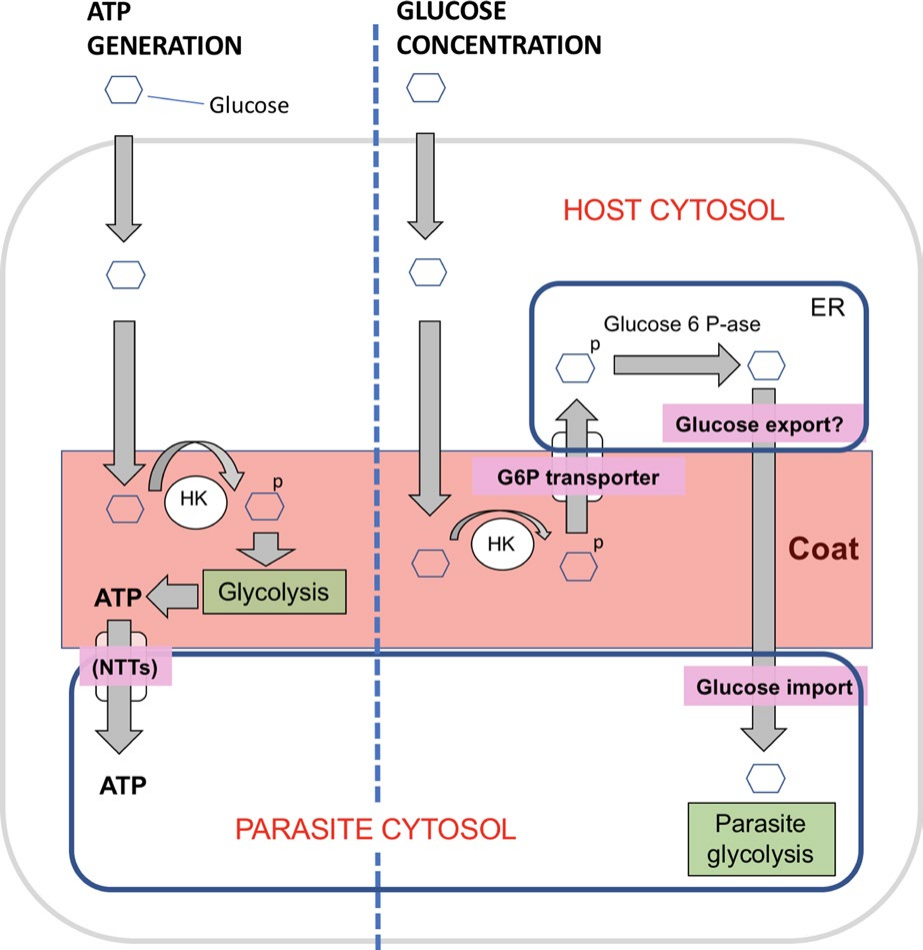
Models of glucose utilization at the *T. hominis*–host cell interface. In the simplest scenario (left), glucose is metabolized by secreted HKs as part of a locally enhanced glycolysis. The ATP molecules generated are taken up into parasites through nucleotide transporters (Heinz et al., 2014). An alternative scheme (right) accounts for the consistent and intimate relationship of host ER with the parasite coat and suggests a more complex model for glucose dynamics. Glucose diffuses into the host cytoplasm and further to the vicinity of the parasite. PQM hexokinases then generate G6P, which is taken up into the ER lumen by transporters present in the ER membrane (G6P/Pi antiporter) and concentrates there. Gluconeogenesis pathways then generate glucose and Pi that are transported out of the ER. This cycle could then increase the concentration of glucose locally for the parasite, either for further entry into a cell surface glycolytic pathway for energy generation (described above) or for direct glucose uptake into the parasite for its own cytosolic glycolysis

A simple ATP uptake model is attractive but does not explain or incorporate the closely associated ER, and as such we now consider two more complex mechanisms. The first involves uptake of G6P into the lumen of the ER, where metabolism could generate glucose for the parasite (outlined in Figure 12). The ER is known to import G‐6‐P through transporters in the membrane and convert it into glucose in a pathway for gluconeogenesis (Hiraiwa, Pan, Lin, Moses, & Chou, 1999). The hexokinase in the PQM‐ER complex would facilitate delivery of G‐6‐P to the ER lumen and enhance glucose delivery to the parasite (although the precise pathway for glucose export from the ER is debated (Csala et al., 2007)). Analogs for glucose transporters are predicted to be present in the *T. hominis* genome.

In the second scenario, G6P, generated by PQM HKs, would again enter the ER but would be metabolized by another ER luminal enzyme, hexose‐6‐phosphate dehydrogenase (H‐6‐PDH). Recently H‐6‐PDH was implicated in an alternative energy pathway (Marini et al., 2016) which is thought to drive ^18^F‐fluorodeoxyglucose accumulation in the ER; which could explain the concentration of 2‐NBDG in our experiments. Importantly, it was shown that silencing H‐6‐PDH expression or its activity not only decreased tracer uptake and glucose consumption but also induced severe energy depletion and decreased NADPH content, without parallel alteration in mitochondrial function. Thus, this work (Marini et al., 2016) suggests a new glucose metabolism that uses ER to generate ATP independently of oxidative phosphorylation and glycolysis—a mechanism which is supported by results on glucose handling after H‐6‐PDH deletion (Rogoff et al., 2007). Interestingly, *T. hominis* was isolated from an infection in skeletal muscle in which the function of ER‐located H‐6‐PDH appears independent of its classical role in the pathway involving steroid biosynthesis (Semjonous et al., 2011). It will be interesting to test whether this pathway is active in the PQM‐ER complex of *T. hominis*.

Finally, in the context of cancer cells, HKs have been implicated in generating the Warburg effect, in which aerobic glycolysis is upregulated in part by HKs that are docked at the mitochondrial porin, VDAC. At this location, the HKs exploit ATP generated in mitochondria to drive glycolysis, with the hypothesized advantages of increased carbon for anabolic pathways (Hsu & Sabatini, 2008). Importantly, we did not find evidence for these effects in global metabolism (Figure [Link], [Link]) and did not detect enrichment of HKs host cell mitochondria using immuno‐EM (Figure S3).

In summary, our results identify an infiltrative cell coat, the PQM, which lies at the interface between the microsporidian *T. hominis* and the mammalian host cytoplasm. The presence of two secreted HK isoforms implicates the PQM in orchestrating metabolic energy theft from the host cell.

## CONFLICT OF INTEREST

The authors state that there is no conflict of interest.

## DATA ACCESSIBILITY STATEMENT

Data supporting this study are provided in the results section and as supplementary information accompanying this paper.
