# Supplementary figures and images for: The invasive cell coat at the microsporidian Trachipleistophora hominis–host cell interface contains secreted hexokinases
Source: Microbiologyopen. 2018 Jul 27;8(4):e00696. doi: 10.1002/mbo3.696 (PMC6460350; doi:10.1002/mbo3.696)

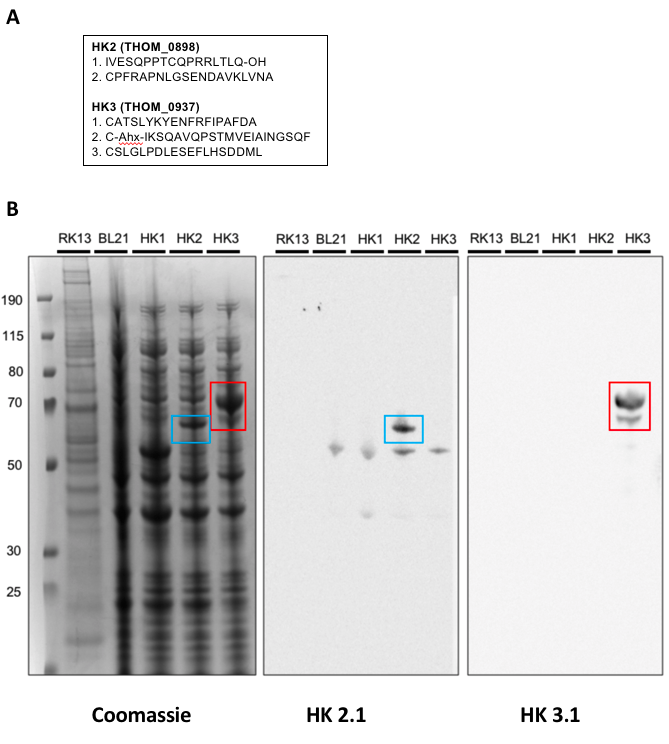

Supplement: Supplementary file 1 [file MBO3-8-e00696-s001.tif]

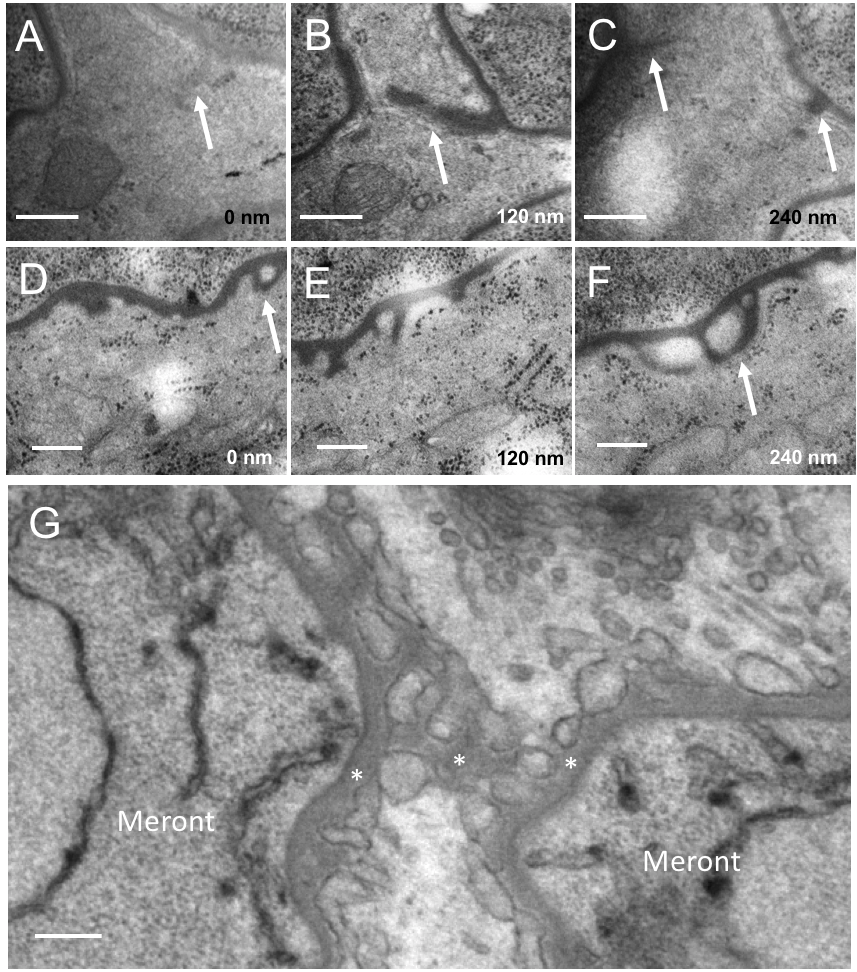

Supplement: Supplementary file 2 [file MBO3-8-e00696-s002.tif]

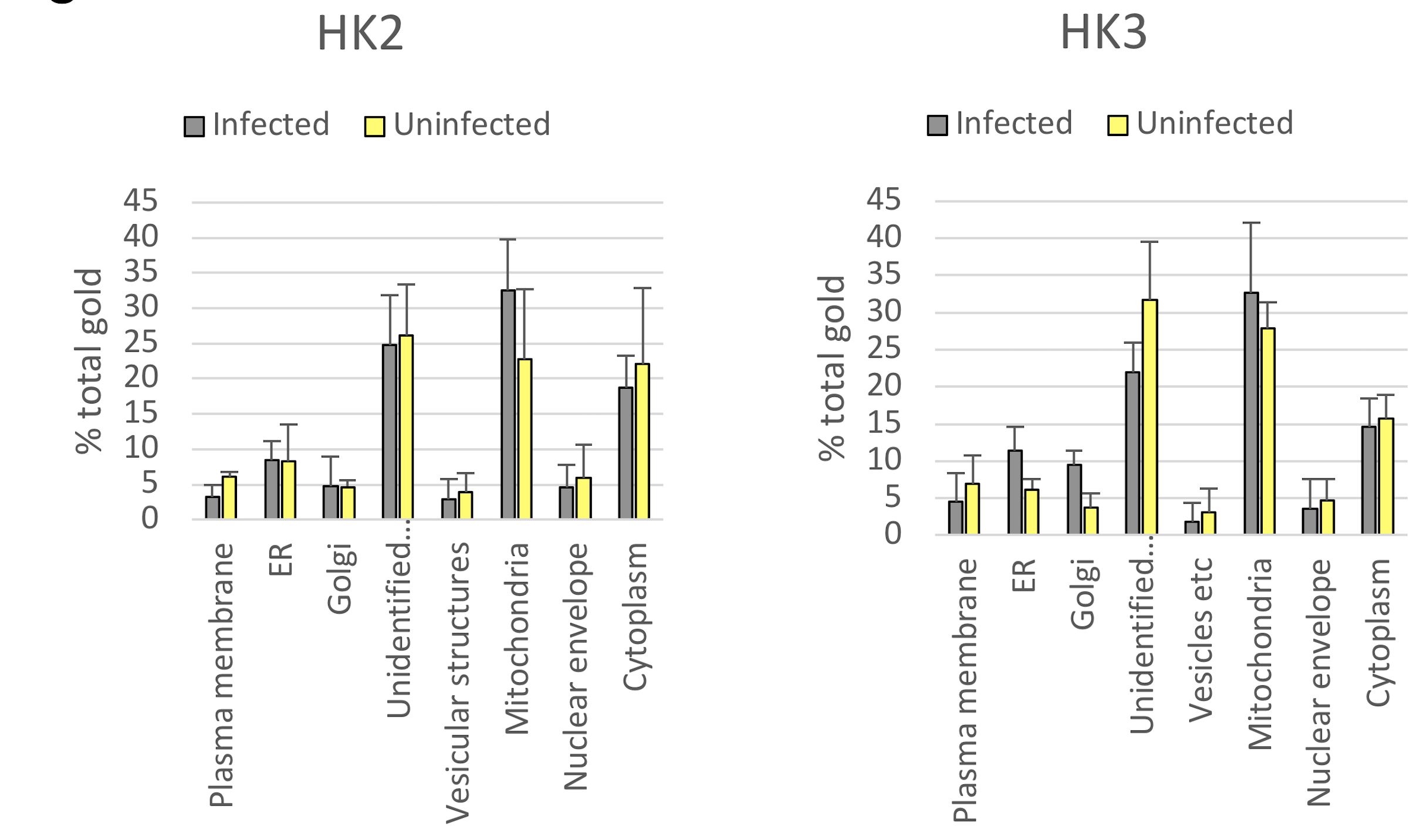

Supplement: Supplementary file 3 [file MBO3-8-e00696-s003.tif]

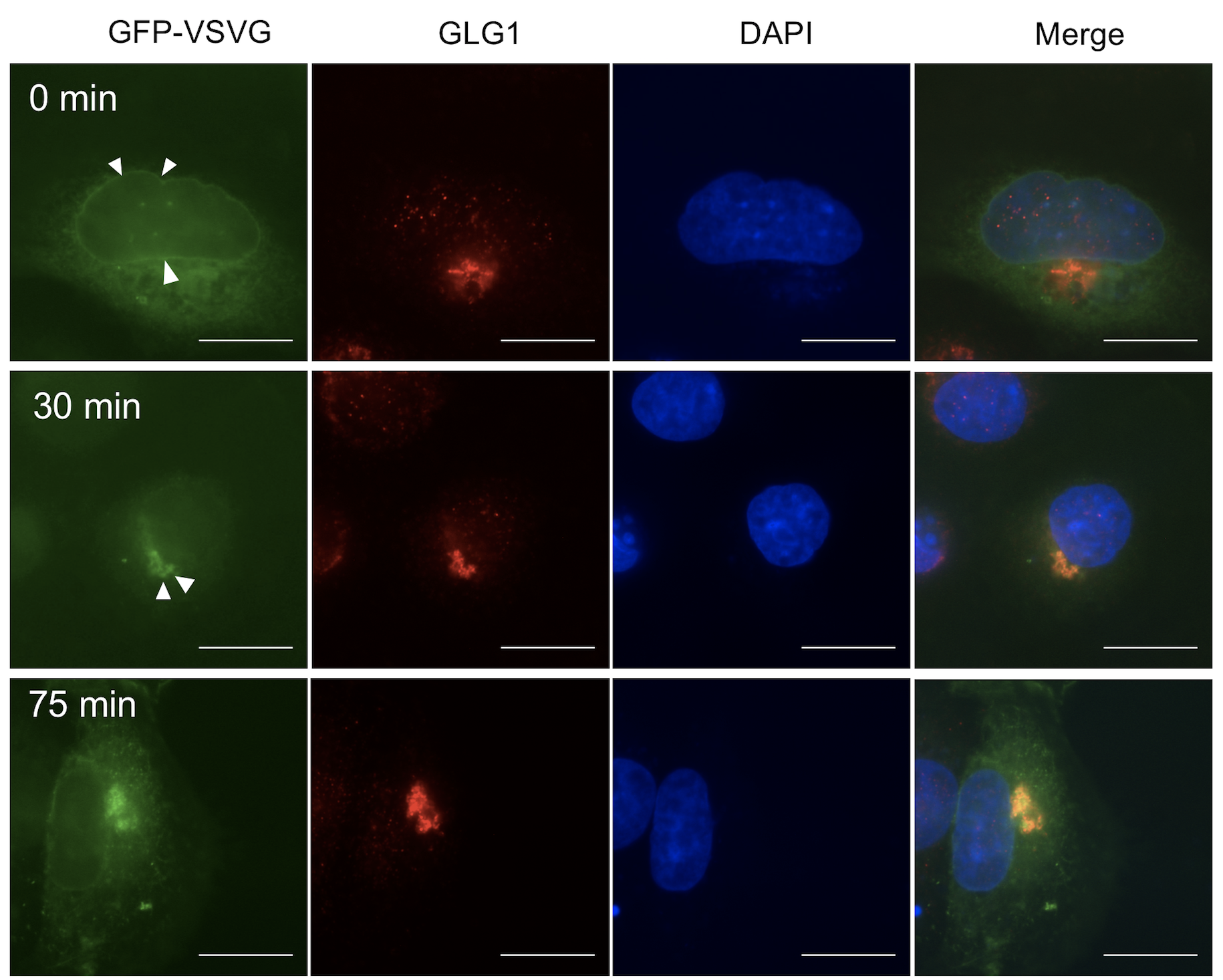

Supplement: Supplementary file 4 [file MBO3-8-e00696-s004.tif]

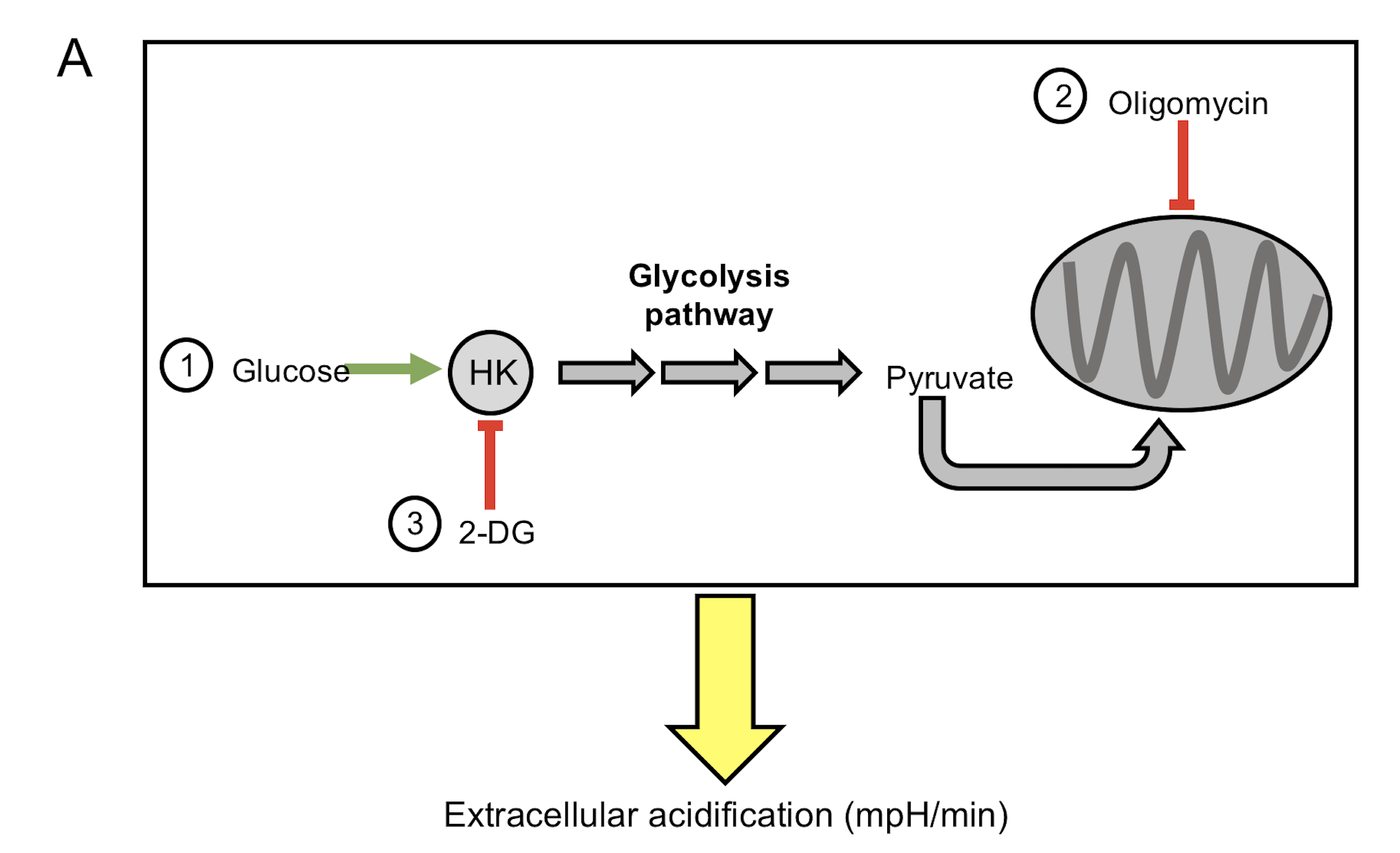

Supplement: Supplementary file 5 [file MBO3-8-e00696-s005.tif]

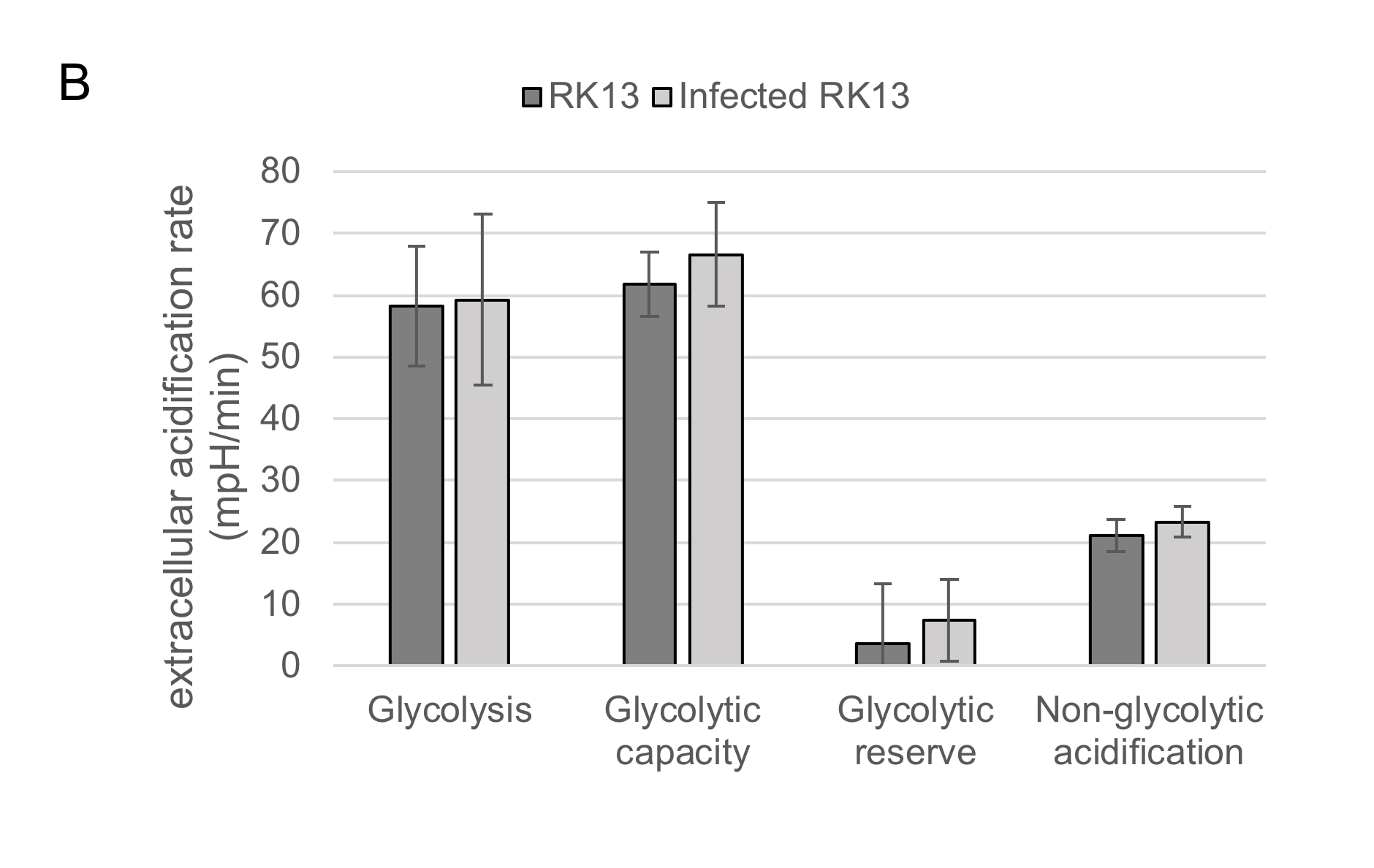

Supplement: Supplementary file 6 [file MBO3-8-e00696-s006.tif]

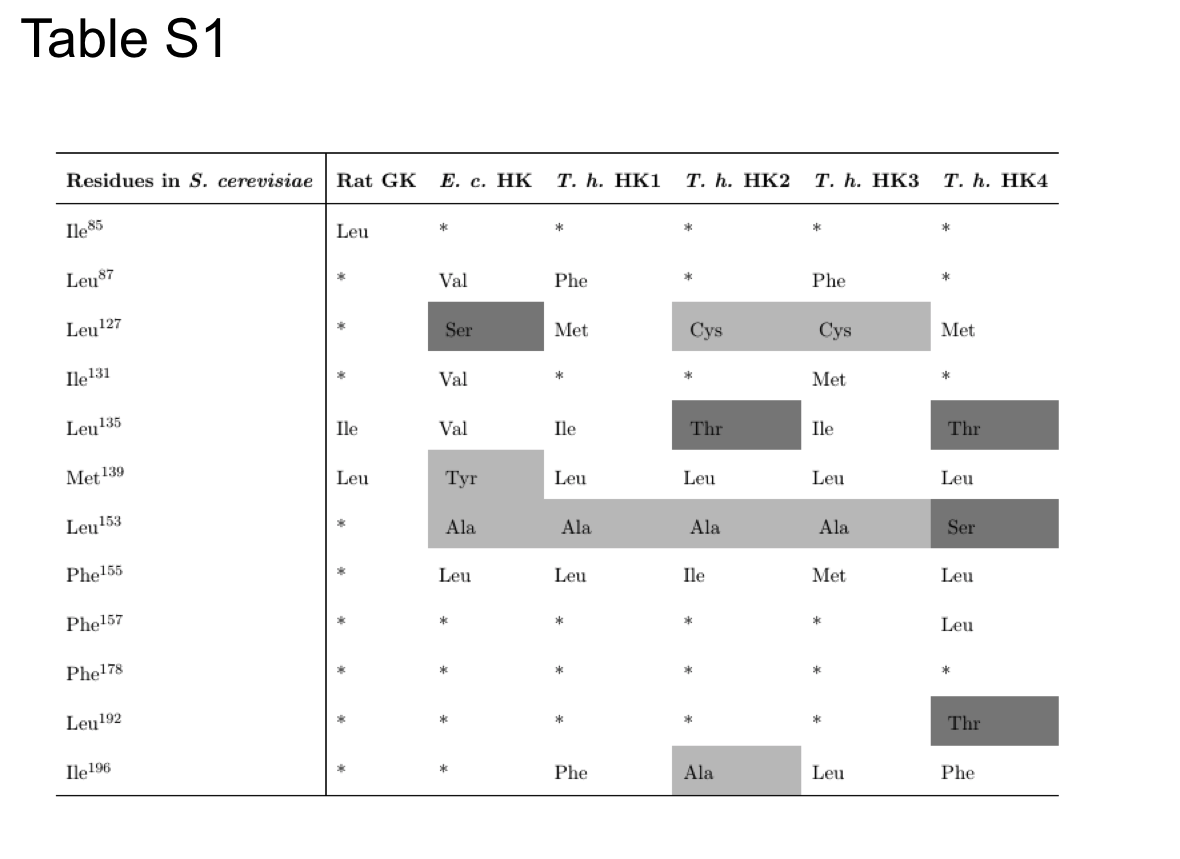

Supplement: Supplementary file 7 [file MBO3-8-e00696-s007.tif]
