# Supplementary material for: The invasive cell coat at the microsporidian Trachipleistophora hominis–host cell interface contains secreted hexokinases
Source: Microbiologyopen. 2018 Jul 27;8(4):e00696. doi: 10.1002/mbo3.696 (PMC6460350; doi:10.1002/mbo3.696)
Supplement: Supplementary file 8 [file MBO3-8-e00696-s008.docx]

Table S2. Gold counts for % gold per stage (HK2; 3 experiments pooled).

| **HK2** | **Surface** | **Membranes** | **Nucleus** | **Cytoplasm** |
| --- | --- | --- | --- | --- |
| **Meront** | 108 | 81 | 9 | 12 |
| **Mature Meront** | 61 | 58 | 1 | 0 |
| **Sporont** | 18 | 7 | 0 | 0 |
| **Spore** | 8 | 0 | 0 | 0 |

Table S3. Gold counts for % gold per stage (HK3; 3 experiments pooled).

| **HK3** | **Surface** | **Membranes** | **Nucleus** | **Cytoplasm** |
| --- | --- | --- | --- | --- |
| **Meront** | 194 | 243 | 15 | 3 |
| **Mature meront** | 87 | 180 | 4 | 1 |
| **Sporont** | 21 | 35 | 0 | 0 |
| **Spore** | 41 | 7 | 0 | 2 |

Table S4. Gold/area quantitation per stage (HK2; 3 experiments separated by commas).

| **HK2** | **total gold** | **total area (µm^2^)** | **gold/area (µm^2^)** | **stages evaluated** |
| --- | --- | --- | --- | --- |
| **Meront** | 91, 39, 113 (235) | 9.84, 10.60, 16.84 | 9.25, 3.68, 6.71 | 18, 12, 21 |
| **Mature meront** | 40, 43, 87 (170) | 2.68, 7.11, 16.33 | 14.92, 6.05, 5.32 | 2, 6, 8 |
| **Sporont** | 31, 9, 38 (78) | 5.84, 2.73, 9.03 | 5.31, 3.30, 4.21 | 4, 2, 6 |
| **Spore** | 2, 57, 21 (80) | 0.81, 14.22, 7.21 | 2.47, 4.01, 2.91 | 1, 8, 4 |
| **HN - infected** | 35, 19, 33 (87) | 35.25, 46.50, 33.53 | 0.99, 0.41, 0.98 | - |
| **HN - uninfected** | 30, 29, 45 (104) | 42.24, 38.95, 49.08 | 0.71, 0.74, 0.91 | - |

Table S5. Gold/area quantitation per stage (HK3; 3 experiments separated by commas).

| HK3 | total gold | total area (µm^2^) | gold/area (µm^2^) | stages evaluated |
| --- | --- | --- | --- | --- |
| Meront | 158, 40, 55 (253) | 9.39, 2.86, 10.51 | 16.83, 13.98, 14.75 | 11, 5, 21 |
| Mature meront | 60, 24, 20 (104) | 5.50, 5.08, 2.25 | 10.90, 4.72, 8.88 | 3, 2, 3 |
| Sporont | 29, 8, 60 (97) | 3.88, 1.39, 5.79 | 7.48, 5.74, 10.36 | 2, 1, 4 |
| Spore | 54, 17, 53 (124) | 5.65, 3.78, 5.23 | 9.56, 4.49, 10.14 | 4, 2, 5 |
| HN - infected | 27, 84, 61 (172) | 37.43, 57.89, 37.43 | 0.72, 1.45, 1.63 | - |
| HN - uninfected | 24, 79, 92 (194) | 24.11, 63.31, 54.30 | 1.00, 1.25, 1.31 | - |

Table S6. Gold over cell coat (HK3, with HK2 meront).

| **Parasite stage** | **Total gold** | **Measurable** | **Tangential** | **Total extensions** | **% tangential** | **% extensions** | **% surface** |
| --- | --- | --- | --- | --- | --- | --- | --- |
| **Meront** | 193 | 72 | 61 | 60 | 31.6 | 31.1 | 68.9 |
| **Mature Meront** | 40 | 33 | 0 | 7 | 0.0 | 17.5 | 82.5 |
| **Sporont** | 68 | 49 | 15 | 4 | 22.1 | 5.9 | 94.1 |
| **Spore** | 39 | 32 | 7 | 0 | 17.9 | 0.0 | 100.0 |
| **Meront (HK2)** | 105 | 49 | 38 | 18 | 36.2 | 17.1 | 82.9 |

Table S7. Gold counts for background host compartments (HK2 and HK3; 3 experiments separated with commas).

|  | **Infected (HK2)** | **Uninfected (HK2)** | **Infected (HK3)** | **Uninfected (HK3)** |
| --- | --- | --- | --- | --- |
| **Plasma membrane** | 5, 4, 1 | 7, 4, 4 | 3, 10, 1 | 11, 3, 10 |
| **ER** | 11, 10, 4 | 14, 6, 2 | 7, 18, 12 | 8, 5, 8 |
| **Golgi** | 3, 2, 7 | 4, 4, 3 | 6, 14, 11 | 6, 3, 3 |
| **Unidentified membranes** | 20, 28, 20 | 26, 14, 22 | 11, 29, 36 | 25, 29, 57 |
| **Vesicular structures** | 4, 0, 4 | 2, 5, 2 | 3, 0, 1 | 7, 1, 2 |
| **Mitochondria** | 49, 26, 21 | 25, 23, 8 | 25, 27, 53 | 31, 28, 34 |
| **Nuclear envelope** | 4, 2, 6 | 2, 8, 3 | 1, 1, 12 | 2, 7, 7 |
| **Cytoplasm** | 24, 21, 10 | 24, 8, 21 | 7, 23, 20 | 13, 18, 22 |
| **Totals** | 120, 93, 73 | 104, 72, 65 | 63, 122, 146 | 103, 94, 143 |
