# Supplementary material for: The invasive cell coat at the microsporidian Trachipleistophora hominis–host cell interface contains secreted hexokinases
Source: Microbiologyopen. 2018 Jul 27;8(4):e00696. doi: 10.1002/mbo3.696 (PMC6460350; doi:10.1002/mbo3.696)
Supplement: Supplementary file 9 [file MBO3-8-e00696-s009.docx]

**Table S1. The hydrophobic channel domain of *S. cerevisiae* HK II, aligned against rat GK and Microsporidial hexokinases.** Peptide sequences contributing to the hydrophobic channel domain in the *S. cerevisiae* HK II were identified by Kuser et al., (Kuser et al. 2000) using crystallographic data. Corresponding residues were analyzed in the following amino acid sequences – *R. norvegicus* glucokinase (GK), *E. cuniculi (E. c.)* HK, *T. hominis (T. h.)* HK1, HK2, HK3 and HK4. Asterisks indicate precise conservation of the residue. No shading indicates conservation of a highly hydrophobic residue. Light grey shading indicates substitution with a less hydrophobic residue. Dark grey indicates substitution with a neutral residue.
