# Supplementary material for: The invasive cell coat at the microsporidian Trachipleistophora hominis–host cell interface contains secreted hexokinases
Source: Microbiologyopen. 2018 Jul 27;8(4):e00696. doi: 10.1002/mbo3.696 (PMC6460350; doi:10.1002/mbo3.696)
Supplement: Supplementary file 10 [file MBO3-8-e00696-s010.docx]

**Table S2 and S3. Immuno-EM HK2 and HK3.** Raw data from Figure 7A with gold counts from scanning band analysis, divided according to parasite stages. To obtain sufficient gold counts for investigating surface labelling of PQM, three experiments were pooled. Table S2 and S3 are data for HK2 and HK3 respectively.

**Table S4 and S5. Intensity of immunogold labelling for HK2 and HK3 (respectively).** Raw data for Figure 7B are expressed as gold/area over the PQM (categorized according to parasite stage) and host nucleus. Data obtained for experiments 1-3 are divided by commas. Bracketed value (Total gold column) is the pooled value of all 3 experiments.

**Table S6. Immuno-EM HK2 and HK3 PQM signal subdivided by tangential, extension and surface gold particles.** Raw data for Figure 8. Tangentially sectioned PQM associated particles (“Tangential”) were observed where no underlying membrane was discernible, thereby PQM could not be classified as surface or extensions. This gold was discounted from analysis.

**Table S7. Immuno-EM scanning band analysis of HK2 and HK3 host compartments.** Raw data for Figure S3. Experiments (1-3) are separated by commas.
